# A high heterozygosity genome assembly of *Aedes albopictus* enables the discovery of the association of PGANT3 with blood-feeding behavior

**DOI:** 10.1186/s12864-024-10133-4

**Published:** 2024-04-03

**Authors:** Yuhua Deng, Shuyi Ren, Qiong Liu, Dan Zhou, Caimei Zhong, Yabin Jin, Lihua Xie, Jinbao Gu, Chuanle Xiao

**Affiliations:** 1 Institute of Translational Medicine Research, The First People’s Hospital of Foshan, #81, North Lingnan Avenue, Foshan, China; 2 State Key Laboratory of Ophthalmology, Zhongshan Ophthalmic Center, Sun Yat-sen University, #7 Jinsui Road, Tianhe District, Guangzhou, China; 3 Department of Pathogen Biology, Institute of Tropical Medicine, School of Public Healthy, Southern Medical University, Guangzhou, China; 4 Key Laboratory of Resource Biology and Biotechnology in Western China, Ministry of Education, Faculty of Life Sciences and Medicine, Northwest University, 229 Taibai North Road, Xi’an, China; 5 Department of Breast Surgery, The First People’s Hospital of Foshan, #81, North Lingnan Avenue, Foshan, China; 6 Department of Dermatology, Shunde District Center for Prevention and Cure of Chronic Diseases, Shunde, China; 7 School of Basic Medical Sciences, Fujian Medical University, No. 1 Xuefu North Road, University Town, Fuzhou, China

**Keywords:** Aedes albopictus, Genome assemble, Hematophagous behavior, PGANT3, Heterozygosity

## Abstract

The Asian tiger mosquito, *Aedes albopictus*, is a global invasive species, notorious for its role in transmitting dangerous human arboviruses such as dengue and Chikungunya. Although hematophagous behavior is repulsive, it is an effective strategy for mosquitoes like *Aedes albopictus* to transmit viruses, posing a significant risk to human health. However, the fragmented nature of the *Ae. albopictus* genome assembly has been a significant challenge, hindering in-depth biological and genetic studies of this mosquito. In this research, we have harnessed a variety of technologies and implemented a novel strategy to create a significantly improved genome assembly for *Ae. albopictus*, designated as AealbF3. This assembly boasts a completeness rate of up to 98.1%, and the duplication rate has been minimized to 1.2%. Furthermore, the fragmented contigs or scaffolds of AealbF3 have been organized into three distinct chromosomes, an arrangement corroborated through syntenic plot analysis, which compared the genetic structure of *Ae. albopictus* with that of *Ae. aegypti*. Additionally, the study has revealed a phylogenetic relationship suggesting that the *PGANT3* gene is implicated in the hematophagous behavior of *Ae. albopictus*. This involvement was preliminarily substantiated through RNA interference (RNAi) techniques and behavioral experiment. In summary, the AealbF3 genome assembly will facilitate new biological insights and intervention strategies for combating this formidable vector of disease. The innovative assembly process employed in this study could also serve as a valuable template for the assembly of genomes in other insects characterized by high levels of heterozygosity.

**Supplementary Information:**

The online version contains supplementary material available at 10.1186/s12864-024-10133-4.

## Introduction

The Asian tiger mosquito, *Aedes albopictus*, is recognized as an aggressive hematophagous insect, playing a critical role as a vector for various serious human diseases. Its presence significantly amplifies public health concerns on a global scale [1–3]. The impact of *Aedes albopictus* on human health is exacerbated by its rapid and aggressive expansion from its native habitat, coupled with its remarkable ecological adaptability across a range of traits, including feeding behavior, diapause, and vector competence [4, 5]. *Ae. albopictus* is a competent vector for at least 26 arboviruses, and it is particularly adept at transmitting diseases such as dengue fever and Chikungunya [6, 7]. It is also implicated as a vector of filarial nematodes of veterinary and zoonotic significance [8, 9]. As a crucial vector, the fragmented genome assembly of *Ae. albopictus* has evidently impeded its biological research.

**Fig. 1:**
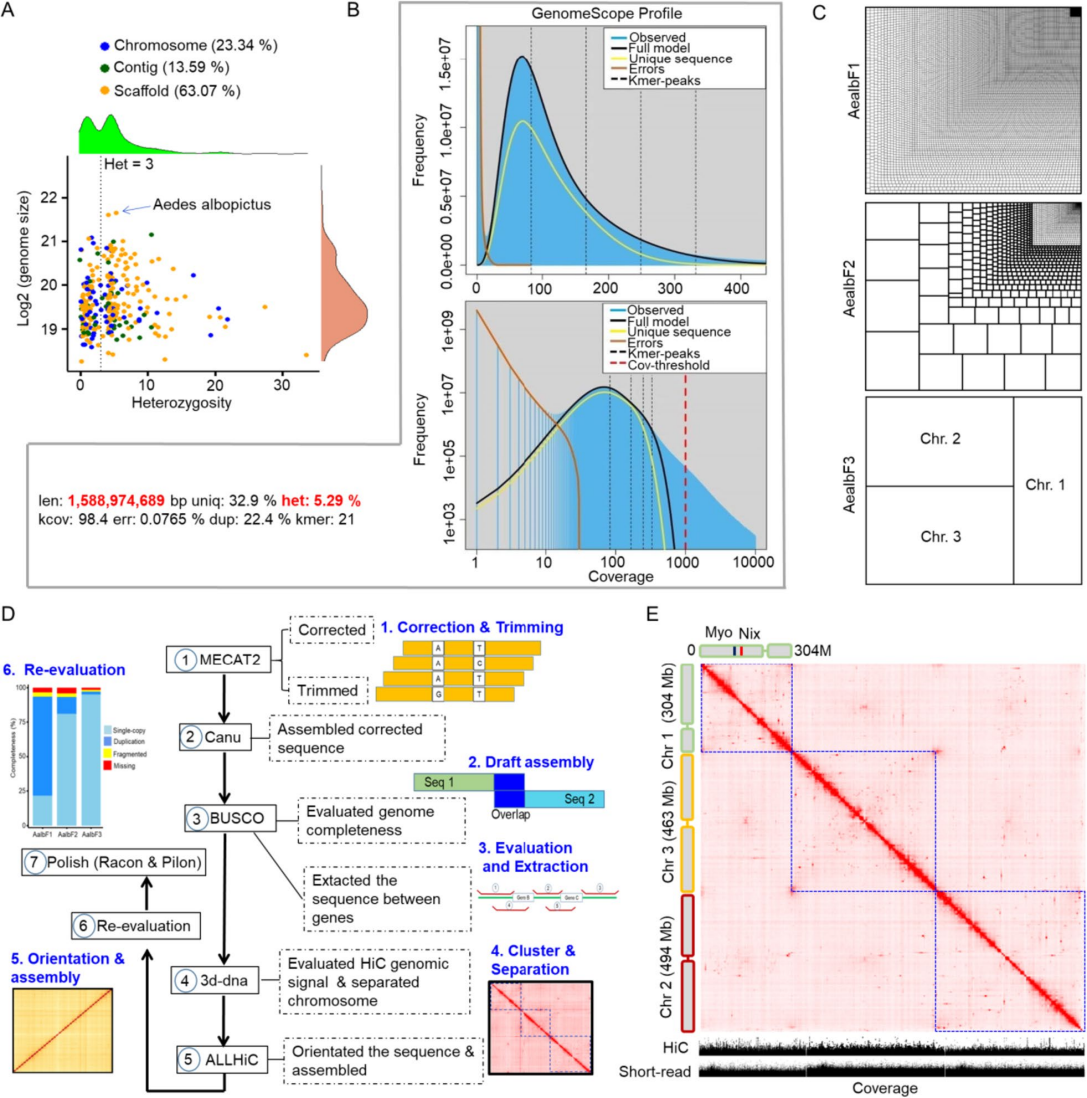
Genomic evaluation and workflow of genome assemble. **a** Heterozygosity prediction for insect genomes. The Dotted line represented heterozygosity levels equivalent to 3. **b** Genomic prediction for the *Ae. albopictus* genome. **c** Treemaps showed the completeness of the *Ae. albopictus* genomes. The length of contigs or scaffolds was plotted. **d** Workflow chart for the newly assembled genome. **e** Clustering of contigs and separation of potential chromosomes of the *Ae. albopictus* genome based on Hi-C alignment. Three chromosomes were identified. The blue square indicated chromosomal boundaries. Chromosomal coverage was determined using Hi-C and Illumine short-reads, respectively. Genes *Nix* and *myo-sex* were utilized to further define chromosome 1

The high heterozygosity of the *Ae. albopictus* genome, estimated at around 5.29% (Fig. 1A), presents a significant challenge in the assembly process. This level of genetic variation can complicate the identification and removal of duplicated sequences, which is a critical step in obtaining a clean and accurate genome assembly. Tools like Purge_haplotigs (v1.1.2) [9], Purge_dups (v1.2.5) [10], and Khaper [11] are designed to help with the removal of duplicated sequences. However, even with these professional packages, the high heterozygosity can make it difficult to effectively filter out duplicated sequences from the *Ae. albopictus* draft genome. To overcome these challenges, it is necessary to employ additional strategies that not only facilitate assembly but also effectively filter out duplicated sequences. In the context of this study, we used a specialized workflow that led to the successful creation of a high-quality genome assembly for *Ae. albopictus*, referred to as AealbF3. This assembly boasts a higher level of completeness compared to previous versions. Additionally, we preliminary verified the role of *PGANT3*, an O-glycosyltransferase, in the hematophagous behavior of *Ae. albopictus*. This is a significant finding as it is the first time that PGANT3 has been linked to the blood-feeding behavior of this mosquito species.

On the other hand, when comparing the newly generated AealbF3 genome with the previously published AealbF1 and AealbF2 genome [12, 13], several improvements have been noted. One of the key advancements is the increased completeness of the assembly, with fragmented sequences now organized into three chromosomes. This more refined assembly allows for the identification of a greater number of non-redundant transcripts. Additionally, the AealbF3 genome shows a high degree of similarity with the previously published *Ae. albopictus* genome in terms of various genomic features, such as the presence and distribution of transposable elements (TEs), the length of coding DNA sequences (CDS), exons, and introns. This consistency across different assemblies suggests that the AealbF3 genome is a reliable reference for further research into the biology of *Ae. albopictus*. The improved quality of the AealbF3 assembly can facilitate more accurate functional annotations, comparative genomics, and evolutionary analyses. It can also aid in the identification of genes and pathways involved in the mosquito’s biology, including those related to its hematophagous behavior. This, in turn, could lead to the development of novel strategies for the prevention and control of this species.

## Results

### Successfully assembled *Ae. albopictus* genome using a special workflow

Predicting the genome size of *Ae. albopictus* using the *C*-value and k-mer analysis is a common approach in genomics. The predicted *C*-value for the *Ae. albopictus* genome ranged from 0.62 to 1.66 picograms (pg), which translates to an estimated genome size of approximate 1.34 to 1.63 gigabases (Gb) [14]. This estimate was highly consistent with the *k*-mer prediction that genome size was 1.588 Gb (Fig. 1B). Our predicted genome size was in line with the results from previous cytofluorimetric studies, which suggested that the genomic haploid length of *Ae. albopictus* was between 1.190 ~ 1.275 Gb [13, 15]. Thus, our prediction of genome size was reasonable.

The impact of genomic heterozygosity on genomic assembly is a significant factor to consider, particularly for species with high levels of genetic variation [16]. In this study, the prediction of heterozygosity across a range of insect genomes revealed that a substantial proportion of these genomes exhibit high heterozygosity (Supplementary Table 1), with over 60% (173 out of 287) showing heterozygosity levels higher than 3 (Fig. 1A). The fact that only about 23.34% (67 out of 287) of these insect genomes had been assembled into the chromosome level (Fig. 1A) underscored the difficulty of assembling genomes with high heterozygosity. The high heterozygosity of *Ae. Albopictus*, estimated at 5.29%, indeed presented a significant challenge for genome assembly. The high number of contigs (509,843, Fig. 1C) in AealbF1 assembly suggested a highly fragmented assembly and indicated a higher duplication rate (~ 76.1%, Fig. 1C and Supplementary Fig. 1C). The AealbF2 assembly, while showing improvements with longer sequences and a lower duplication rate (Fig. 1C), still did not reach the chromosomal level. Therefore, a high-quality genome assembly would accelerate biological and genetic researches, and the improved reference genome for *Ae. albopictus* became indispensable.

We initially processed approximate 627 Gb of Pacbio long reads (418× coverage) using MECAT2 with default parameter [17], obtaining ~ 167 Gb of high-quality and clean reads, which corresponds to approximate 111 times coverage (111×). These cleaned reads was then assembled into a draft genome using four different assembly packages. The consistency of results from each package suggested that the assembly process was robust across different algorithms (Supplementary Fig. 1B and Supplementary Table 3, upper). The observation that the duplicated pattern of single-copy genes (SCGs) from the BUSCO dataset was almost identical (Supplementary Fig. 1B), with approximate 82.9% SCGs being duplicated at least two times (Supplementary Fig. 1C), indicated a high level of duplication in the genome of *Ae. albopictus*. However, the difficulty in completely removing genomic duplication, despite the use of professional packages like Purge_haplotigs (v1.1.2) [18], Purge_dups (v1.2.5) [10], and Khaper [11], highlighted the challenges associated with assembling genomes with high heterozygosity and duplication rates. The implication that conventional assembly procedures might not be suitable for the newly assembled *Ae. albopictus* genome (AealbF3) suggested that new strategies or improvements may be necessary.

The observation that SCGs might act as “traffic hubs” connecting flanking sequences around them suggested a complex interplay between gene duplication and the surrounding genomic context (Supplementary Fig. 1D). Meanwhile, BLAST results from BUSCO estimation showing different alignment scores for the same SCG across diverse contigs or scaffolds implied a high degree of sequence variation within the genome, which might induce duplication (See “Method and material” section). Based on our deduction, flanking sequences (> 15,000 bp) around duplicated SCGs with higher BLAST alignment scores were extracted. Following a special workflow (Fig. 1D), Hi-C data were aligned to the extracted flanking sequences, and the best Hi-C contact map (0.hic) from 3d-dna (v180922) [19] was used to separate potential chromosomes (Supplementary Fig. 2). Finally, using ALLHiC (v0.9.8) [11], the flanking sequences clustered into potential chromosomes were further orientated and assembled. The successful assembly of the AealbF3 genome into three chromosomes, with lengths of approximate 304 million base pairs (Mbp) for chromosome 1, 494 Mbp for chromosome 2, and 463 Mbp for chromosome 3, represents a significant advancement in the genomics of *Ae. Albopictus* (Fig. 1E and Supplementary Fig. 3). The result was consistent with flow cytometry results described by Palatini U et al. [13], and along with the anticipated genome size of approximate 1.23 GbThe high coverage (~ 90%) of the three chromosomes by both Hi-C (high-resolution chromosome conformation capture) and Illumina short-read data (Fig. 1E), as well as the further definition of chromosome 1 by the location of two specific genes, *Nix* (the dominant male-determining factor, MN364861.1) and *myo-sex* (myosin heavy chain protein, XM_019707039.1), provided strong evidence for the preliminary completion of the chromosome-level assembly in the AealbF3 genome (scaffold N50: 463.2M) using our specialized workflow.

### Evaluation and annotation of the newly assembled AealbF3

**Fig. 2:**
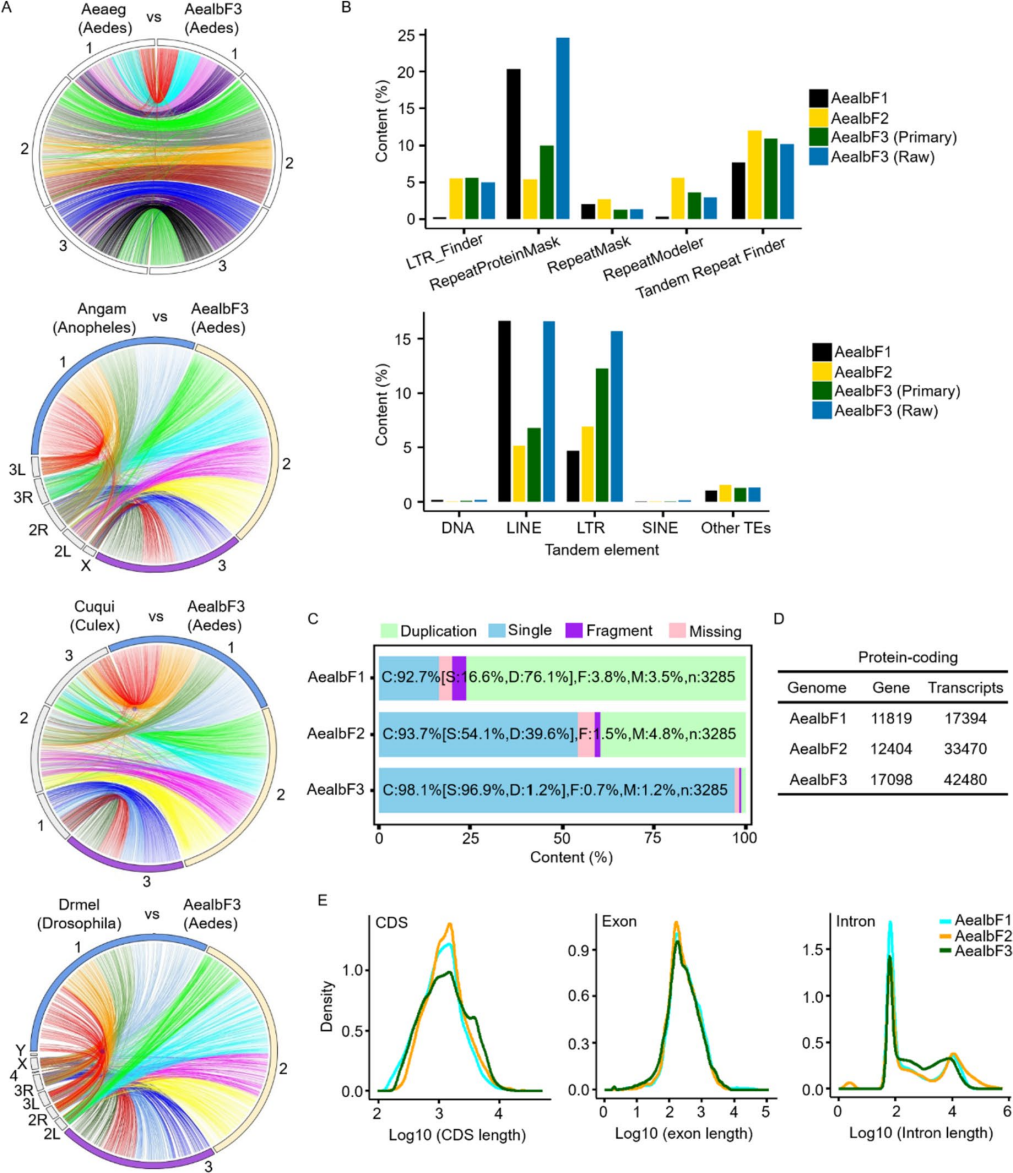
Evaluation and annotation of the newly assembled AealbF3 genome. **a** Protein sequences from AealbF3 was aligned with those of *Ae. aegypti* (Aeaeg), *An. gambiae* (Angam), *Cu. quinquefasciatus* (Cuqui), and *Dr. melanogaster* (Drmel) to assess the genic location of AealbF3. Syntenic blocks are linked between genomes in a circos plot. The chromosomal labels were shown around the circos. Brackets indicate the genus of each genomes. **b** Comparison of tandem element to confirm the genomic assembly. Similar results were observed among AealbF1, AealbF2, and AealbF3. LINE, long terminal repeat retrotransposon; SINE, short interspersed nuclear element; DNA, DNA transposon; LTR, long terminal repeat. “Raw” refers to the draft assembly; “Primary” indicates the removal of contigs lacking single-copy genes from the BUSCO dataset. **c** Evaluation of genome completeness using BUSCO. **d** Comparison of protein-coding genes and transcripts between AealbF3 and those of AealbF1 and AealbF2. **e** Comparison of CDS length, exon, and intron length among AealbF1, AealbF2, and AealbF3

The assessment of the AealbF3 genome assembly’s accuracy through comparison with other dipteran species such as *Aedes aegypti*, *Drosophila Melanogaster*, and *Culex quinquefasciatus*, had demonstrated its high quality and completeness. The syntenic plots constructed using homologous genes indicated a well-assembled genome, particularly in the comparison with *Ae. aegypti* (Fig. 2A). Comparative genome alignment analyses further supported this, showing a reasonable degree of homogeneity, with the closest relationship observed between *Ae. albopictus* and *Ae. aegypti*, and the most distant between *Ae. albopictus* and *D. melanogaster* (Supplementary Fig. 4). The consistency in the contents of tandem repeat elements (TE), such as DNA transposons and SINE, across different versions of the *Ae. Albopictus* genome, despite variations in duplication ratios, also suggested a reliable assembly (Fig. 2B, upper panel). Slight variations observed in LINE and LTR elements (Fig. 2B, lower) were noted but do not significantly impact the overall quality.

The use of BUSCO (v3.0.2) with the diptera_odb10 dataset, which includes 3285 SCGs, further validated the completeness of the AealbF3 genome assembly. Compared to previous versions AealbF1 and AealbF2, AealbF3 showed a higher level of completeness (98.1%), with lower rates of duplication (~ 1.2%) and missing genes (~ 1.2%) (Fig. 2C). This improved assembly had enabled the identification of more non-coding RNAs (Supplementary Table 5) than in our previous study (AealbF1) [12]. Gene annotation analysis revealed that AealbF3 contained approximate 17,098 protein-coding genes (including 42,480 non-redundant transcripts (Fig. 2D). The average lengths of CDS, introns, and exons were 1,828.69 bp, 2,941.9, and 465.73 bp, respectively. These values were comparable to those of AealbF1 and AealbF2 (Fig. 2E). Moreover, 99.56% of the CDS were complete, and the completeness of protein sequences in AealbF3 was comparable to that of AealbF2 but superior to AealbF1 (Supplementary Fig. 5). This indicated that the AealbF3 assembly not only maintained gene quality but also provided a richer set of transcripts for further research, which is crucial for understanding the biology of *Ae. Albopictus* and its role as a vector for diseases.

### Identification of positively selected genes associating with dipterous hematophagous behavior

**Fig. 3:**
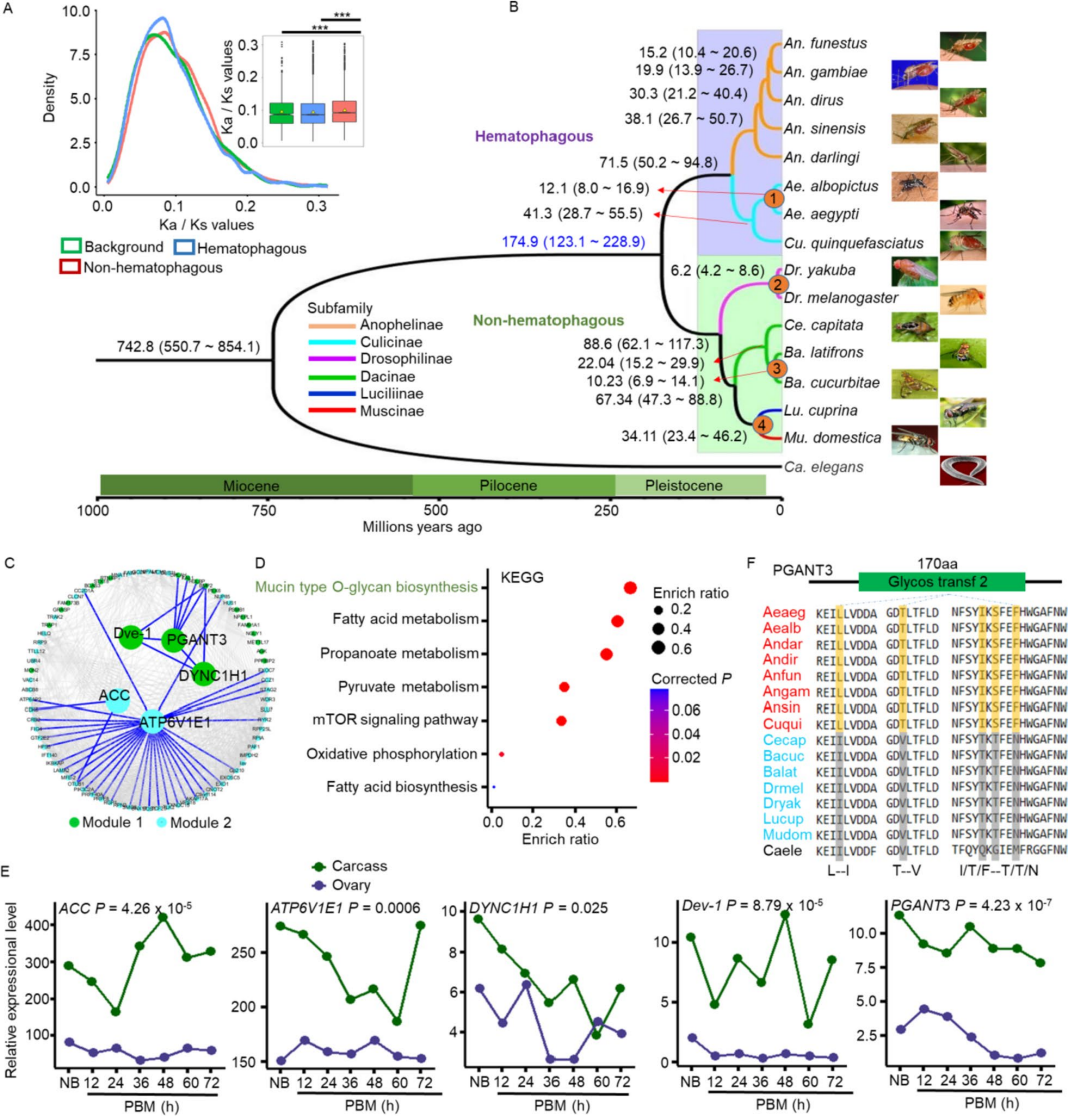
Phylogenetic prediction of positively selected genes. **a** *Ka*/*Ks* values were used to predict selection pressures. The results were calculated from blocks of whole-genome duplicated alignments. **b** A phylogenetic tree with divergence time calibration constructed using orthologous genes. Four fossils from Timetree (www.timetree.org/) were utilized to infer divergence times. The possible separation time between hematophagous and non-hematophagous species is highlighted in blue. Fossils was numbered and marked at the nodes. Colored branches represented the same subfamily. **c** Identification of critical positively selected genes. Genes were clustered into two co-expression modules colored in darkgreen and cyan, respectively. **d** The KEGG pathway of five critical positively selected genes are shown. Pathway colored in darkgreen represented module 1, while black one was module 2. **e** Differential expression of five critical PSGs between carcass and ovary was presented. **f** Mosquito-specific mutation in the gene *PGANT3* was highlighted. Specific mutation was highlighted and showed under the sequence. Red label represented mosquitoes, while cyan label represented flies. Caele was an outgroup colored in black. Protein domains were colored in darkgreen

The hematophagous behavior of *Ae. albopictus* is a crucial foundation for the transmission of diseases that threaten human health [20, 21]. However, the evolutionary and molecular basis of this behavior might not be entirely clear. Due to the conservation of mitochondrial DNA, we compared the phylogenetic trees of single-copy homologous genes and mitochondria, predicting that hematophagous and non-hematophagous dipterous insects likely underwent evolutionary separation between 174.9 and 86.1 million years ago (Myo) during the Pleistocene epoch (Fig. 3B and Supplementary Fig. 6). The evolution pressure, represented by the *Ka*/*Ks* value, indicated a significant difference between hematophagous and non-hematophagous dipterous insects (Fig. 3A and Supplementary Fig. 7A), implying that these two groups had experienced diverse selective pressure [22].

Consequently, we identified 93 positively selected genes (PSGs) in the hematophagous branch using PAML (v4.9) (Supplementary Fig. 7). To focus on the PSGs that might be more critical for dipterous hematophagous behavior, we employed the WGCNA algorithm (Weighted Gene Co-expression Network Analysis) and identified five critical PSGs: *ACC* (acetyl-CoA carboxylase), *ATP6V1E1* (ATPase H + transporting V1 subunit E1), *DYNC1H1* (dynein heavy chain), *PGANT3* (α-N-acetylgalactosaminyltransferases 3), and *Dve-1* (homeobox protein dve-1), which were involved in two separated co-expressional modules (Fig. 3C and Supplementary Table 6). Module 1, containing *Dve-1*, *PGANT3*, and *DYNC1H1*, was primarily associated with the biosynthesis of mucin-type O-glycan (module names: M00056) in the golgi apparatus and endoplasmic reticulum (Fig. 3D and Supplementary Fig. 7B), which was involved in serine and threonine metabolism [23, 24]. This pathway is one of the most important posttranslational modifications of proteins, participating in protein conformation, sorting, developmental processes, and modulation of enzymatic activities, as verified in vertebrates and invertebrates [25–27]. Additionally, module 2, containing *ACC* and *ATP6V1E1*, is primarily involved in metabolic pathway such as fatty acid, propanoate, and pyruvate metabolism (Fig. 3D and Supplementary Fig. 7B). The higher expression of these genes in female carcasses (Fig. 3E) suggested they may be associated with some physiological function preceding a blood meal.

However, mosquito-specific mutations are likely critical drivers of hematophagous behavior. We investigated whether the five critical PSGs contained mosquito-specific mutations in their protein domains. After comparison with the Pfam database, we found at least five typical mosquito-specific mutations in the “Glycos transf 2” domain of PGANT3 (Fig. 3F). Similar findings were also observed in the protein domains of other genes, such as *ATP6V1E1*, *ACC*, and *DYNC1H1* (Supplementary Fig. 8). Collectively, these findings suggested that the five genes with mosquito-specific mutations may be associated with hematophagous behavior of mosquitoes.

### Silenced *PGANT3* impacted on mucin type O-glycan biosynthesis

**Fig. 4:**
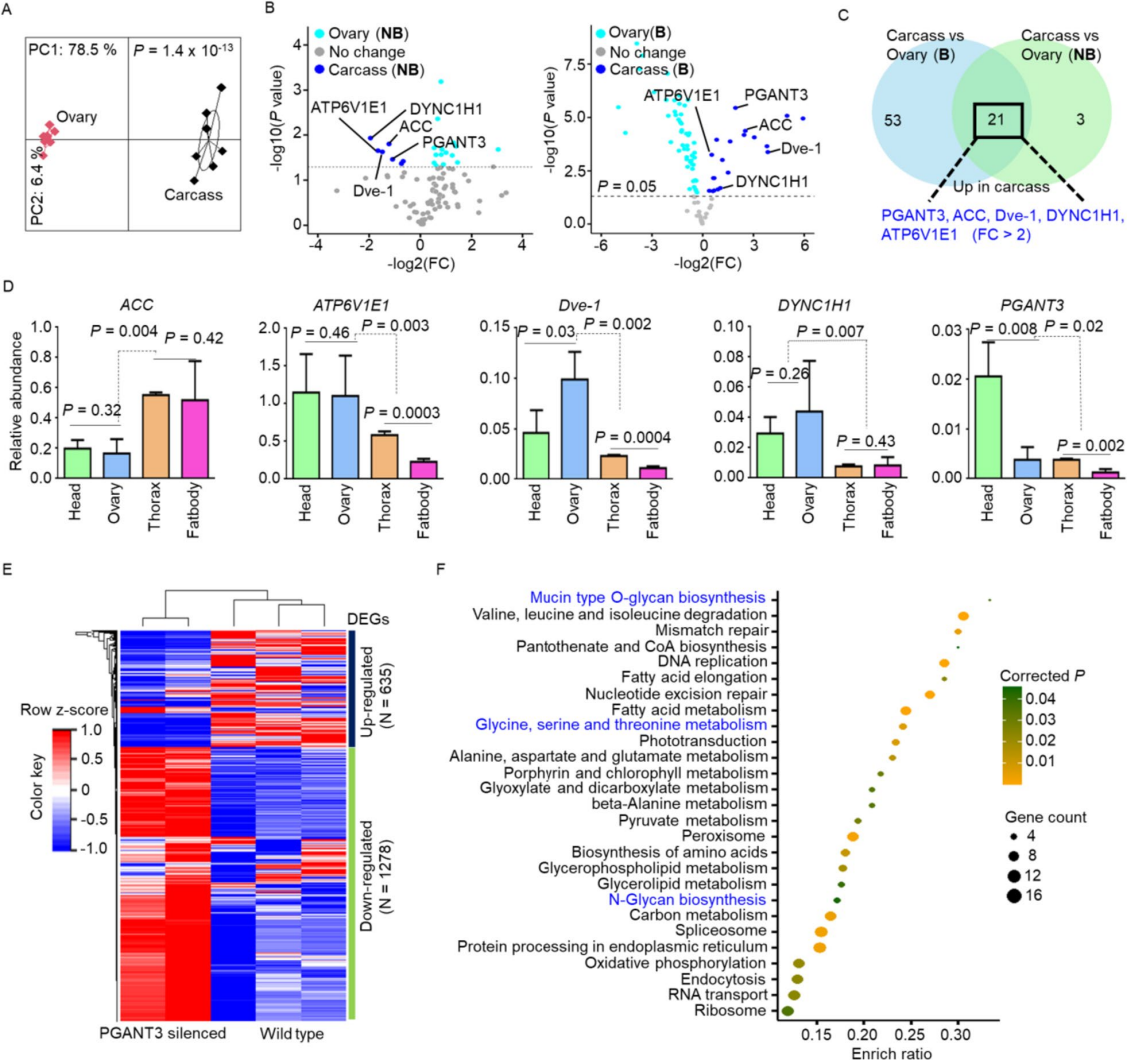
PGANT3 was associated with O-glycan biosynthesis. **a** Principal component analysis was conducted to compare the expression levels between ovaries and carcasses. **b** Volcano plots showed the differential expression between carcasses and ovaries. Five critical PSGs were labeled and the horizontal dashed line showed a *p*-value threshold of 0.05. “NB” denotes non-blood-fed mosquitoes, while “B” denotes blood-fed mosquitoes. **c** Venn plot indicated the common PSGs that were unregulated in the carcasses. Only genes with fold change > 2 were showed and colored in blue. **d** Tissue-specific expression of five PSGs using real-time PCR. Relative abundance of gene was compared with the reference gene RpS7. **e** Heatmap plot showed the RNAi results of differential expression genes. Total RNAs of mosquitoes after dsRNA-PGANT3 injection were performed RNA-sequencing, while wild-type ones were used as control. **f** KEGG analysis of down-regulated genes after RNAi. Pathways involved in O-glycan biosynthesis were colored in blue

The α-N-acetylgalactosaminyltransferases PGANT3 plays a significant role in the biosynthesis of mucin-type O-glycan and regulated O-linked glycosylation (GalNAcα1-O-S/T) [28], which is crucial for posttranslational modifications of proteins. These modifications are important for protein conformation and developmental processes [29, 30]. Additionally, O-glycan are required for normal nervous system development and function [31, 32], as well as cell-cell communication [33, 34]. Given these roles, we investigated the impact of *PGANT3* on the hematophagous behavior of *Ae. albopictus*. Our initial findings showed a significant difference in PGANT3 expression between carcasses and ovaries (Fig. 4A), and among the five previously identified PSGs, only PGANT3 was upregulated at least twofold in both blood-fed and non-blood-fed carcasses (Fig. 4B, C). To determine the specific tissues expressing these PSGs, we performed real-time RT-PCR and found that ACC was primarily expressed in the thorax and fat body, while *ATP6V1E1*, *Dve-1* and *DYNC1H1* were more highly expressed in the head and ovaries (Fig. 4D). Notably, the *PGANT3* expression was significantly increased in the head (Fig. 4D).

Further investigation into the function of *PGANT3* through RNAi experiment and RNA-seq revealed that silencing *PGANT3* led to the downregulation of 1278 genes (Fig. 4E). These genes are involved in O- or N-glycan biosynthesis and influence protein processing in the endoplasmic reticulum (Fig. 4F), aligning with our earlier prediction (Fig. 3D and Supplemental Fig. 7B). Additionally, related metabolic pathways, such as fatty acid, pyruvate, and amino acid metabolism, were also attenuated (Fig. 4F). The spliceosome, which affects mRNA alternative splicing and protein diversity [35], was also weakened (Fig. 4F). These results suggested that *PGANT3* was likely involved in mosquito behavior related to host-seeking by influencing O-glycan biosynthesis and protein function.

### Silenced *PGANT3* weaken *Ae. albopictus’*s hematophagous behavior

Mosquito brains are known to drive host-seeking behavior, as described in studies on vampire bats [36]. We could conceptually divide the hematophagous behavior of *Ae. albopictus* into two stages, similar to the blood-feeding process of vampire bat [37]: the “consciousness stage”, which drives host-seeking before the blood meal, and the “digestion stage”, which involves assimilating blood protein after blood meal. Genes involved in the consciousness stage are typically expressed in the brain or eye, while those involved in digestion stage are primarily found in the liver, intestines, stomach, or other digestive organs [37]. Therefore, the high expression of *PGANT3* in the head of *Ae. albopictus* suggested it might influence hematophagous behavior during the consciousness stage.

**Fig. 5:**
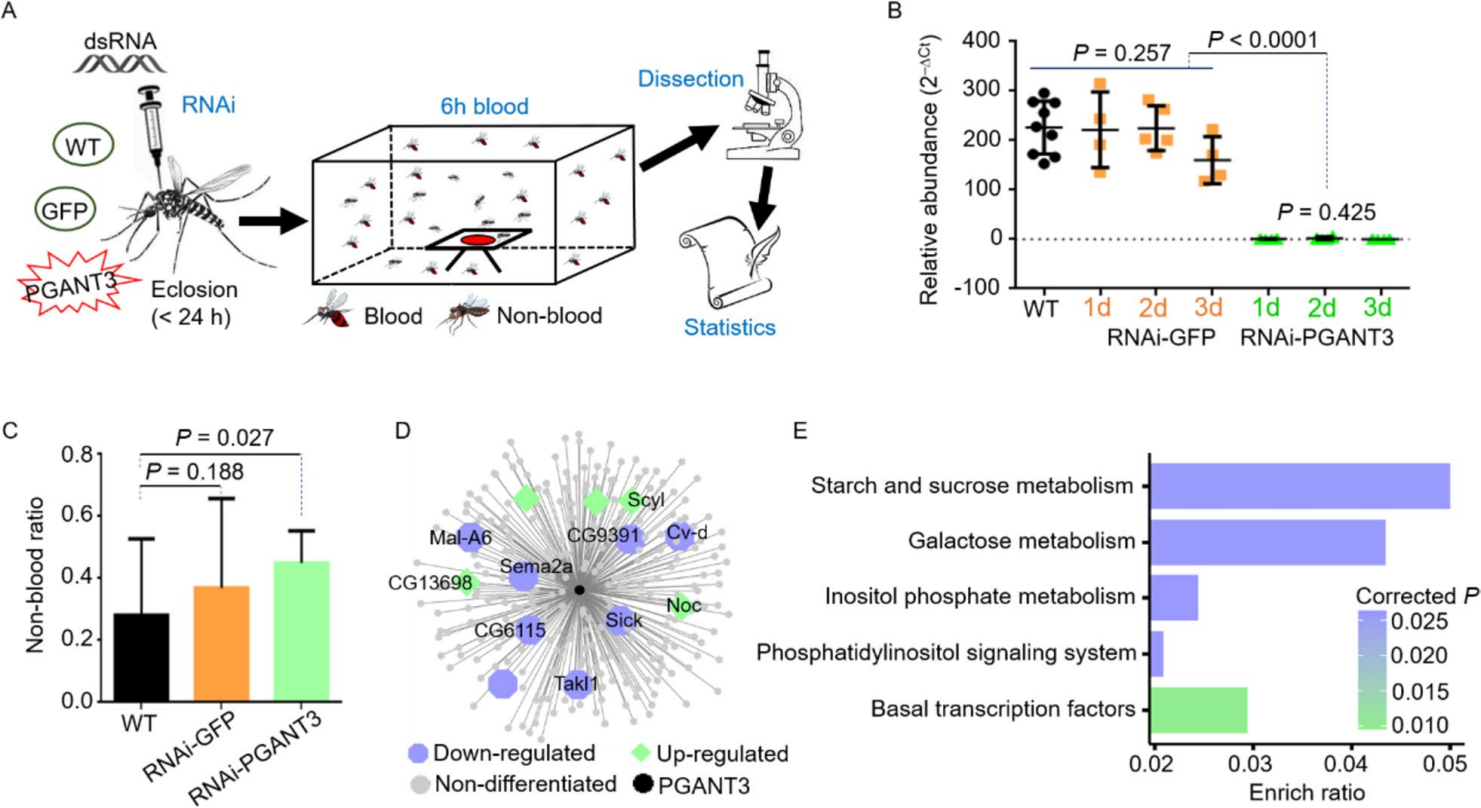
Functional evaluation of *PGANT3* after RNAi through *Ae. albopictus’* hematophagous behavior. **a** workflow indicated the experiment of blood meal. **b** Real-time RT PCR detected the expression of *PGANT3* after RNAi. **c** Statistics of RNAi-mosquito after blood meal. **d** WGCNA showed the relative genes of *PGANT3* after RNAi. **e** Metabolic pathway of relative genes after ds-PGANT3 interference. Color was consistent with Fig. 5d

To test this hypothesis, we conducted a behavioral experiment to estimate to estimate the blood-feeding behavior of mosquitoes injected with dsRNA targeting PGANT3 (ds-*PGANT3*), in comparison to wild-type (WT) and sham (dsRNA-GFP) groups. The detailed workflow was shown in Fig. 5A. After RNAi, the expression level of *PGANT3* was significantly reduced (Fig. 5B). Interestingly, the blood-feeding behavior of mosquitoes in the ds-*PGANT3* group was also notably weakened (Fig. 5C, WT: 0.2818 ± 0.0569, dsRNA-GFP: 0.3692 ± 0.0669, ds-*PGANT3*: 0.4499 ± 0.0241). This reduction in blood-feeding behavior might be associated with attenuated pathways, such as starch, sucrose, and galactose metabolism (Fig. 5D, E). Collectively, these results suggested that *PGANT3* was likely associated with the hematophagous behavior of *Ae. albopictus*, supporting the initial prediction.

## Discussion

An accurate and complete genome assembly is essential for understanding the unique aspects of mosquito biology and for developing control strategies to reduce their capacity to spread pathogens [31, 38]. Fragmented genome assemblies have historically made it challenging to deeply investigate the biological functions of genes, which is crucial for formulating more effective prevention and control strategies against *Ae. albopictus*. The high genome heterozygosity of *Ae. albopictus* is a significant factor that has impeded genome assembly efforts. In this study, we leveraged various assembly software and implemented a specialized workflow to successfully generate a new, more complete genome assembly for *Ae. albopictus*, designated as AealbF3. Despite some minor translocations identified through Hi-C assembly, we have provided a method to address assembly challenges in insects with high heterozygosity. Using AealbF3, we compared homologous genes between hematophagous and non-hematophagous dipterous insects and identified a gene, *PGANT3*, in mosquitoes. While previous studies had shown that PGANT3 is an O-glycosyltransferase involved in biosynthesis of O-glycosylation and impacts cell adhesion during Drosophila development [28–30, 39, 40], its role in mosquito hematophagous behavior was not well understood. Therefore, we conducted preliminary experiments to investigate the function of *PGANT3* in relation to *Ae. albopictus*’s hematophagous behavior. These findings require further verification and may offer new avenues for exploring the biology of *Ae. albopictus* and developing strategies to mitigate its impact on public health.

## Method and material

### Text 1. Species information

The study utilized 15 dipteran genomes, which included 8 hematophagous mosquito species and 7 non-hematophagous fly species (Supplementary Table 2). The mosquito species comprised *Aedes albopictus* (with one newly assembled genome AealbF3 and two previously published genomes AealbF1 and AealbF2, specifically the Chinese Foshan strain), *Aedes aegypti*, *Anopheles darlingi*, *Anopheles dirus*, *Anopheles sinensis*, *Anopheles funestus*, *Drosophila melanogaster*, *Anopheles gambiae*, *Drosophila yakuba*, *Culex quinquefasciatus*, *Ceratitis capitate*, *Musca domestica*, *Lucilia cuprina*, *Bactrocera cucurbitae*, and *Bactrocera latifrons*. *Caenorhabditis elegans* was used as an outgroup for comparison. The complete mitochondrial genomes of these species can be accessed through the Eukaryota Mitochondrial Genomes Database (https://www.ncbi.nlm.nih.gov/genomes/).

### Text 2. Do novo genome sequencing, assembly, annotation and evaluation of new AealbF3 genome

#### Mosquitoes

The *Ae. albopictus* strain utilized in this study was originally isolated in 1981 from the wild in Foshan County, Guangdong Province, China, and was sourced from the Guangdong Provincial Center for Disease Control and Prevention (CDC). The mosquitoes were maintained under standard insectary conditions, which included a temperature of 27 ± 1 °C, relative humidity ranging from 70 to 80%, and a photoperiod of 14 h light followed by 10 h of darkness. The larval stage of the mosquitoes was reared in pans, fed with a diet consisting of finely ground fish food mixed with yeast powder in a 1:1 ratio. Adults mosquitoes were provided with a carbohydrate source in the form of a cotton wick soaked in 0.2 g/ml sucrose solution. Adult females mosquitoes were given the opportunity to feed on blood meals using a blood-soaked pledget technique approximate 3 ~ 4 days after emerging from their pupal stage. The blood meals contained commercially available defibrinated sheep blood mixed with 1% sucrose, as described in our previous study [41].

#### Sample collection, DNA extraction, genome sequencing

In our previous study (AealbF1), DNA was extracted from a single pupa to perform sequencing [12]. The DNA for the recently published genome AealbF2 was sourced from pupae of inbred single pair mosquitoes [13]. However, the level of heterozygosity and duplication did not show significant change, indicating that the gender of pupae was not associated with heterozygosity and genomic duplication during assembling. In the current study, we collected a total of 100 live pupae, washed them three times in sterile water, and extracted at least 30 µg of genomic DNA manually following the provided instructions. Two libraries ware prepared with average length of 20 Kb and 40 Kb, respectively. Whole-genome sequencing was conducted using Single Molecule Real-Time (SMRT) sequencing technology from Pacific Biosciences, USA. The sequencing libraries were prepared using the SMRTbell Template Prep Kit 1.0 and sequenced on a Pacific Biosciences Sequel instrument. The sequencing was carried out by Beijing BerryGenomics Co., Ltd (Beijing, China), resulting in approximate 627 Gb of raw long sequences in FASTA formal. Additionally, DNA was digested with DpnII, and Hi-C data, approximate 1030 Gb in FASTQ formal, were generated using pair-end sequencing with a read length of 150 bp.

#### Prediction of genome size

##### C-value

The *C-value* refers to the total amount of DNA present in a single set of chromosomes within a cell. To covert the *C-value* into base pairs, which represent the genome size, the conversion factor of 1 pg of DNA being equivalent to 978 Mbp is commonly used. According to the genome size database (www.genomesize.com), the *C-value* for the *Ae. albopictus* genome, as determined from sperm, ranges from 0.62 to 1.66 pg. Using the conversion factor, this would translate to an estimated genome size of approximate 1342 ~ 1638 Mbp (since 0.62 pg * 978 Mb/pg = 605.76 Mbp and 1.66 pg * 978 Mb/pg = 1624.48 Mbp). Additionally, it has been verified that *Ae. albopictus* has a diploid chromosome count of 6, which means that the genome is composed of these six chromosomes [14].

##### k-mer analysis

The Illumina shortgun DNA reads, which have an average length of 90 bp, were obtained from a single pupa DNA sample in our previous study [12]. These reads were utilized for *k*-mer frequency analyses. A *k*-mer is a subsequence of a DNA sequence consisting of a specific number of consecutive nucleotides, denoted as ‘k’. In this context, *k*-mers are used to analyze the sequencing reads by dividing them into segments of ‘k’ bases. The frequency of each *k*-mer can be determined from the genome sequence reads, and *k*-mer analysis can be employed to estimate genome size using the formula G = *k*_num/*k*_depth, where G represents the genome size in gigabases (Gb), *k*_num is the total number of unique *k*_mers, and *k*_depth is the average coverage depth of these *k*_mers. In this analysis, different *k*-mers sizes (such as k = 17, 19 and 21) were used, and the calculations were performed using Jellyfish (v2.3.0) [42] and GenomeScope [43]. The results showed that the relationship between *k*-mer frequency and sequence depth follows a *Poisson* distribution, which is a common pattern observed in sequencing data. Based on this analysis, the estimated haploid genomic size of *Ae. albopictus* ranged from 1.245 Gb to 1.588 Gb.

#### Calculation of heterozygosity

The genome data for the selected species were obtained from the Sequence Read Archive (SRA) database, as detailed in Supplementary Table 1. The raw sequencing data were processed using the FASTQ-dump tool from the SRAtoolskit (v2.11.2) [44] to extract FASTQ files. To ensure data quality, low-quality reads and adaptor sequences were filtered out using Cutadapt (v1.9.1) with the following parameter: -q 20 -m 60 --trim-n -O 5 [45]. This step helps to remove reads with a Phred quality score below 20 and trims reads to a minimum length of 60 bases after removing any N’s (ambiguous bases) from the ends of the reads. Subsequently, duplicated reads and reads containing more than 10% N’s were filtered using PRINSEQ (v0.0.12) with the parameter: -derep 1 and -ns_max_p 10, respectively [46]. The -derep 1 parameter is used to remove duplicated reads, while -ns_max_p 10 sets the maximum proportion of ambiguous bases allowed in a read. The resulting clean reads in FASTQ format were then converted into FASTA format using FASTOOL (v0.1.4) [47]. This conversion is necessary for subsequent analysis using tools like Jellyfish and GenomeScope, which are employed to estimate genomic heterozygosity.

#### Genome de novo assembly

Based on the predicted genome size and heterozygosity, we planned to use four different assembly tools: MECAT2 [17], Canu [48], Wtdbg [49], and Flye [50], to assemble the genome. The raw long reads were corrected and trimmed using MECAT2 with default parameters, resulting in approximate 167 Gbp (111×) of high-quality and clean reads. These clean reads were then assembled using the four assembly packages with their default parameters. Despite the high heterozygosity, the assembly results were quite similar across the different packages: the estimated genome size was around 4.5 Gbp, the GC content was approximate 40.4%, the completeness was about 97%, and the duplication rate was around 82.9% (Supplementary Table 3, upper).

Using BUSCO (v3.0.2) [51], we identified single-copy genes (SCGs) in the dataset, which are distributed throughout the genome and serve as a measure of genomic completeness. We considered contigs or scaffolds containing SCGs as the primary sequence in the draft genome. After filtering, the newly draft genomes were re-estimated, and similar results were found for completeness, duplication, and GC content (Supplementary Table 3, lower). The genome size was in perfect agreement with our prediction, especially the results from Canu, which contained the most accurate genome size and the longest contigs (Supplementary Table 3, lower). The newly filtered draft genome from Canu was then further assembled into chromosome.

However, regardless of the assembly package used, we found that approximate 82.9% of SCGs were embedded in at least two or more contigs or scaffolds (Supplementary Fig. 1B, C). This suggested that SCGs may act as “traffic hubs” connecting flanking sequences (Supplementary Fig. 1D). Another possibility is that the same SCGs have different sequence similarities between contigs or scaffolds, which was comfirmed by BLAST results from BUSCO (as is full_table.tsv).

To address this, flanking sequences (> 15,000 bp) around SCGs with higher BLAST scores were extracted. These sequences, along with other contigs or scaffolds without duplicated SCGs, were aligned with Hi-C data using BWA (0.7.17-r1188) [52], and the mapped SAM file (mapping ratio: 85.14%) was processed with Juicer (v1.7) [53]. The merged_nodups file from Juicer was then used to generate a Hi-C heatmap using 3d-dna (v180922) with default parameters [19]. The de-duplicated and unpolished genome yielded a better Hi-C contact map (0.hic, Supplementary Fig. 2), which was visualized in Juicerbox (v1.11.08) [54]. Utilizing ALLHiC for orientation [11], the contigs of scaffolds (> 15,000 bp) in potential chromosome were separated and assembled. For further correction, single nucleotide polymorphism and indels (insertions and delections) were polished with Racon (v1.5.0) [55] using long reads and Pilon (v1.24) [56] using shortgun reads. The assembly workflow was divided into six steps: 1) Correction and trimming of raw reads; 2) Assembly of clean reads; 3) Evaluation and extraction based on BUSCO evaluation; 4) Clustering contigs or scaffolds into potential chromosome according to the Hi-C contact map using 3d-dna; 5) Orientated and assembly of potential chromosomes using ALLHiC; 6) Re-evaluation; and 7) Polished. This comprehensive approach aimed to improve the quality and accuracy of the genome assembly, particularly for species with high heterozygosity like *Ae. albopictus*.

#### Evaluation of genome completeness with BUSCO

BUSCO (v3.0.2) was widely used to assess the genome completeness of newly assembled genomes of *Ae. albopictus*, as well as all selected insect genomes described in this study. It estimated the percentage of expected single copy conserved orthologs, referring to the diptera_odb10 BUSCO set. Additionally, *Ca. elegans*, serving as an outgroup, was evaluated using nematoda_odb10 dataset.

#### Genome annotation

##### Repeat annotation

We initially employed LTR_FINDER (v1.0.6) [57] and RepeatModeler (v1.0.11) [58] for de novo prediction of repeats. Subsequently, RepeatMasker (v4.0.7, with the parameters: -nolow -no_is -norna -parallel 20) [59] was used to identify known transposable elements (TEs) by searching against the Repbase (v16.10) [60]. This approach was applied for DNA-level identification based on homology. Tandem repeats across the genome were then identified using Tandem Repeats Finder (TRF v4.09, with settings: 2 7 7 80 10 50 500 -h -d) [61]. Additionally, RepeatProteinMask software (v4.0.7, with parameters: -noLowSimple -*p*value 0.0001) was utilized to identify proteins element related to TEs. The various categories of repeat elements are depicted in Fig. 2B.

##### Identified of non-coding RNA

Insect genomes were aligned with the Rfam database [62] to identify potential non-coding RNAs (ncRNAs) using INFERNAL (v1.1.2) [63] with default parameters. The outputs from INFERNAL were filtered based on an E-value threshold of less than 0.05 to ensure the significance of the matches. The ncRNAs, including tRNA, snRNA, C/D box snoRNA, miRNA, and rRNA, were identified through this process (Supplementary Table 5).

##### Gene annotation

To predict the protein-coding genes of the *Ae. albopictus* genome (AealbF3), we employed a combination of homology-based, ab initio prediction methods, and transcriptomic data. The quality of the data was assessed using FastQC (v0.11.5) [64]. To ensure alignment accuracy across all reads, we initially used Cutadapt (v1.9.1, with parameters: -q 20 -m 50 –trim-n -O 5) to filter out potential adapter sequences and low-quality reads. The automatic eukaryotic genome annotation was carried out using Braker2 (v2.1.6) [65], which utilized homology proteins from various species, including *Ae. albopictus*, *Ae. aegypti*, *Dr. melanogaster*, *An. gambiae*, and *Cx. quinquefasciatus*. Following this, low-quality sequences, such as those with short lengths (< 50 aa), low percent identity (< 25%), and prematurely terminating genes, were excluded from the consensus gene set. Redundant sequences were further removed using CD-HIT with parameters: -c 0.85 -d 0 -T 20 -aS 0.8 [66]. Lastly, low-quality gene locations were filtered using GFF3Clear.pl script. The package ANGEL was utilized to evaluate the completeness of the annotated gene into three categories: completeness, 3’ partial, and 5’ partial. This comprehensive approach ensures a high-quality gene annotation that can be used for further functional analysis and comparative genomics studies.

##### Whole genome synteny

Genomic syntenic relationships between the AealbF3 genomes and those of *Ae. aegypti*, *An. gambiae*, *Cu. Quinquefasciatus*, and *Dr. melanogaster* suggested that the AealbF3 genome is well-assembled. Initially, protein sequences from each species were aligned with those of *Ae. albopictus* using diamond (v2.0.14) [67]. Alignments with at least 80% identity were retained, and a circos plot was generated using TBtools (v1.0987671) [68].

To further confirm these genomic syntenic relationships, we identified syntenic blocks using MUMmer4 (v4.0.0rc1) [69]. Each genome was aligned to the AealbF3 genome using “nucmer” command with the parameters: --threads 32 --mum -D 5. Subsequently, the “delta-filter” command was applied to the output.delta file to create an output.best.delta, using the parameters: -i 60 -l 500 -1 -o 70, to refine the syntenic blocks. Finally, the “best.delta” file was converted into a PNG-format file using mummerplot (v3.5) with the parameter: -t png.

### Text 3. Phylogenetic analysis and gene families calculation

#### Phylogenetic tree construction

##### Mitochondrial tree

We initially used the mitochondrial genome sequence, which was downloaded from Eukaryota mitochondrial genomes database (https://www.ncbi.nlm.nih.gov/genomes/GenomesGroup.cgi?opt=organelle&taxid=2759), to construct phylogenetic tree. MUSCLE (v3.8.31) [70] with default parameters was employed to perform the alignment, and the resulting PHYLIP-format alignment was further used to estimate the tree using RAxML (v8.2.12, with parameters: -f a -N 1000 -x 12,345 -p 12,345 -m GTRGAMMA) with 1000 bootstrap replicates [71]. The best tree, with the maximum likelihood (ML) value, was selected and is shown below:

- ((Anodir:0.02708640910277367031,(((Anodar:0.07171013819654634136,Anosin:0.04330055845067067727):0.01167026592036247458,(((Luccup:0.07072473540443263706,Musdom:0.08478027589104210338):0.05083344426647806030,((Dromel:0.04598054077149545116,Droyak:0.03814999952770091463):0.09445567733971520219,((Baccuc:0.09076329966439963814,Baclat:0.12696118996287286684):0.05422553669688405520,Cercap:0.07320567119813506385):0.08181666907864505733):0.02154268557325686798):0.17183869488311212526,(Aedalb:0.08454669041529314089,(Culqui:0.06617818908814639134,Aedaeg:0.03833484278876195944):0.02439059847325333180):0.04520042781239510393):0.04900769936872210325):0.00731589533520718947,(Anofun:0.06563534419406583109,Anogam:0.05927470386085681442):0.01036247115248725009):0.03719538367087871916):6.58159522014559783543,Caeele:6.58159522014559783543);

##### Orthologous gene tree

We retrieved protein-coding sequences for 15 dipteral species (Supplementary Table 2) and one outgroup (*C. elegans*) from the NCBI database. For each gene model with multiple isoforms, the longest sequence was selected to perform an all-against-all search algorithm to identify orthologous gene. A total of 551 single-copy orthologous gene groups were identified using OrthoFinder (v2.3.7, with parameters: -t 40 -a 1000 -S blast -I 1.5) [72]. These groups were aligned using MUSCLE and concatenated into supergene sequences using in-house Perl scripts. A PHYLIP-format alignment file containing all single-copy orthologous genes was then generated. The supergene sets were further used to estimate a phylogenetic tree using RAxML (v8.2.12, with parameters: -f a -N 1000 -x 12,345 -p 12,345 -m GTRGAMMA) with 1000 bootstrap replicates. The tree with the maximum likelihood (ML) value was selected as the best tree and is showed below:

- (((((Cercap:0.11978024798387100491,(Baclat:0.06696957820661558758,Baccuc:0.05632958289609005936):0.07336417104858808380):0.16841258689236715540,(Luccup:0.17511956663335589979,Musdom:0.18385908353407340310):0.14021734839310945331):0.17178379806079713266,(Droyak:0.03527297194386659618,Dromel:0.03512750913665669411):0.24882860791157671021):0.29954859069716599507,(((((Anofun:0.13351390587030512180,Anogam:0.08476925479208108349):0.02822623896493825155,Anodir:0.09213358565104126652):0.04318593994596637425,Anosin:0.13994779989026320810):0.03108203873331032499,Anodar:0.16948983534538120121):0.12275033401636810393,(Culqui:0.14922185359818096462,(Aedaeg:0.08909641128962357548,Aedalb:0.07166413443289476604):0.12555362049699445026):0.07870745821588201463):0.31406745566655314139):2.55904117738638259283,Caeele:2.55904117738638259283);

#### Calculated of *Ka/Ks*

Pairwise alignment blocks from whole genome alignments were utilized to calculate the *Ka*/*Ks* ratio to predict evolutionary pressure. Self-alignments of each insect genome were performed using LAST (v992) with the parameter settings “lastal -E 0.05 index_name fasta-sequence-file > result.maf” [73]. The resulting MAF-format files were then converted into AXT-format using the maf-convert script. Furthermore, syntenic blocks in AXT-format (> 1000 bp and with an identity < 99%) were used to calculate *Ks* values (synonymous substitutions per synonymous site) using KaKs_Calculator (v2.0) [74]. We compared the *Ka*/*Ks* ratios among dipteral species, with the outgroup serving as a reference. The results indicated that the selective pressure in hematophagous species differed from that of non-hematophagous species (Fig. 3A). A *Ka*/*Ks* > 1 indicated positive selection, *Ka*/*Ks* = 1 represented neutral selection, and *Ka*/*Ks* < 1 signified purifying selection.

#### Divergence time calibration

We utilized the mitochondrial tree and orthologous gene trees described above to calibrate divergence times. The best tree was selected for estimating divergence times using the MCMCTREE program within PAML (v4.9), employing Bayesian approaches [75]. Five fossils calibrations estimated by Timetree (http://www.timetree.org/) were incorporated, including those for *Ba. latifrons* and *Ba. cucurbitae* (73 Mya, 22 ~ 86 Mya), *Lu. cuprin* and *Mu. domestica* (55 Mya, 47 ~ 71 Mya), *Cu. quinquefasciatus* and *Ae. aegypti* (150 Mya, 2 ~ 174 Mya), *Ae. albopictus* and *Ae. aegypti* (37 Mya, 20.3 ~ 57.2 Mya), and *Dr. yakuba* and *Dr. melanogaster* (6.2 Mya, 4.2 ~ 8.6 Mya).

### Text 4. Genomic features related to dipterous hematophagous evolution

#### Identification of positively selected genes (PSGs)

We employed orthologous genes to identify PSGs, applying a series of rigorous filtering criteria: (1) the longest isoform was selected; (2) frame-shift indels within the CDSs were removed; (3) CDSs containing premature stop codons were excluded; and (4) genes with *K*s values exceeding two in the identified gene groups were excluded. After these filtering steps, a total of 7,553 orthologous genes groups were retained. The tree topology depicted in Fig. 3B served as the prior tree topology. To estimate the lineage-specific evolutionary rate for each branch, we ran the Codeml program from the PAML package (v4.9) with the free-ratio model (model = 1) for each orthologous group.

Positive selection signals on genes along specific lineages were detected using the optimized branch-site model. A likelihood ratio test (LRT) was performed to compare a model that permitted positive selection at sites on the foreground branch with a null model allowing for neutral and purifying selection at these sites. The *p*-values were calculated using the Chi-square statistic, and genes with *p*-valuees < 0.05 were considered as positive candidates. We identified 93 common PSGs at the hematophagous branch (Supplementary Fig. 7A). Functional enrichment analysis of these PSGs was conducted using KOBAS, with a false discovery rate (FDR) correction applied to the *p*-values, setting a threshold of FDR < 0.05.

#### Identification of mosquito specific mutations

Protein sequences of selected PSGs were compared against the Pfam-A database using Pfam with default parameters (v1.6) [76] to ascertain whether the mutations were located within protein domain. Due to variations among the different selected genomes, some sites in the protein sequences might be missing. A specific amino acid substitution was defined as the mosquito-specific if the amino acids in the mosquito differed from those of all other species, and only if at least 13 species (> 80%) had valid amino acid information. We identified four PSGs (*ACC*, *ATP6V1E1*, *PGANT3*, and *DYNC1H1*) with mosquito-specific mutations in protein domains (Fig. 3F and Supplementary Fig. 8).

### Text 5. Analysis of gene function related to dipterous hematophagous behavior

#### Transcriptomic data processed

Transcriptomic data obtained from RNA interference (RNAi) experiments were preprocessed to remove low-quality reads. Initially, low-quality data were filtered using Cutadapt (v1.9.1) with the parameters: -q 20 -m 60 --trim-n -O 5 [45]. Subsequently, duplicate reads and reads containing more than 10% ambiguous bases were filtered using PRINSEQ (v0.0.12) with the parameters: -derep 1 and -ns_max_p 10, respectively [46]. The cleaned reads were then aligned to the newly assembled AealbF3 genome using tophat2 (v2.1.0) [77]. Read counts were calculated using FeatureCounts (v2.0.3) [78], and differential expression analysis was performed using DESeq2 [79]. All plots were generated by R (v4.2.1).

#### Weighted gene co-expression network analysis (WGCNA)

The WGCNA is commonly used to investigate gene networks with similar expression patterns, which can also help to focus on critical genes [80]. The expression matrix of PSGs was further analyzed using WGCNA to identify co-expression modules. A soft-threshold of 12, determined by mean connectivity, was applied, and modules with high correlation were identified (Supplementary Fig. 9). The connectivity among genes within each module was visualized using Cytoscape (v3.7.1) [81], and critical genes were identified using the Cytohubba package within Cytoscape.

#### RNA interference (RNAi) and real-time PCR

The gene *PGANT3* encodes α-N-acetylgalactosaminyltransferases, enzymes that regulate synaptic O-linked glycosylation (GalNAcα1-O-S/T). The loss of PGANT3 can influence neurotransmission strength and suppress activity-dependent facilitation, augmentation, and posttetanic potentiation in Drosophila [28]. Additionally, *PGANT3* regulats posttranslational modifications of proteins, affecting protein conformation and cell adhesion [29, 30]. Due to these reasons, it is appropriate to perform RNA interference to avoid affecting mosquitoe growth after gene knockout.

Complementary DNAs containing the T7 promoter sequence for PGANT3 were used to synthesize dsRNA using the MEGAscript T7 Kit (Ambion, USA). Green fluorescent protein (GFP) dsRNA served as a control. At least 800 ng of newly synthesized dsRNA was injected into 24-hour-old adult female mosquitoes through the intersegmental thoracic membrane using a drawn-out capillary (1 mm o.d.) with a 40 mm tip aperture connected via Teflon tubing to a 50 ml syringe mounted to a syringe pumTotal RNA was extracted from individual mosquitoes at 2 days post-injection, as this was the time of strongest interference efficiency (Fig. 5B). Residual DNA was removed using RNAase-free DNAase I treatment. The dsRNA primers with T7 promoter (underline) are showed below:

- GFP-T7-F:
- GGATCCTAATACGACTCACTATAGGAATGGGCACAAATTTTCTGTCAGT
- GFP-T7-R:
- GGATCCTAATACGACTCACTATAGGCCGGACTTGTATAGTTCATCCATGC
- Pgant3-T7-F:
- GGATCCTAATACGACTCACTATAGGCAGCGAAGCGTTAGAAGTAGC
- Pgant3-T7-R:
- GGATCCTAATACGACTCACTATAGGATGCTTACCACAACCGGAAG

Triplicate real-time PCR experiments were conducted using miScript SYBR^R^ Green PCR Kit (QIAGEN, Valencia, CA) and the products were analyzed on an MX3005P™ Real Time PCR System (Stratagene, La Jolla, CA, USA). Results were analyzed using the 2^−ΔΔCT^ method. The amplification conditions were as follows: initial denaturation at 95 °C for 10 min, followed by 40 cycles of 95 °C for 15 s, 62 °C for 30 s, and 72 °C for 30 s. The *Ae. albopictus* rpS7 gene was served as a house-keeping control. The PCR primers for five PSGs were shown below:

- PGANT3-F: CGTTGGACATTACGTGGGGA
- PGANT3-R: GCAGCAGATCTTTCGCTTGG
- ACC-F: TTCCGGACGGATACCTCTGT
- ACC-R: ACGGAAGCAATCTGCTGTGA
- ATP6V1E1-F: ATGTCGTTGTCACCCTGGAC
- ATP6V1E1-R: ACCTTGATGCGAGACGACTG
- Dve-1-F: TTTCCTGCTTTGCGAAGTGC
- Dve-1-R: TCGGTACGGGCCTTCTTTTC
- DYNC1H1-F: GGCTCGCCAAATCGACAATC
- DYNC1H1-R: GGACAGTTTGGCACGGAAAC
- RpS7-F: ATGAACTCGGACCTGAAG
- RpS7-R: TTCTTGCTGTTGAACTCG

#### Behavior experiments

To further investigate the function of *PGANT3*, we conducted blood-feeding experiments using female mosquitoes that had been injected with approximate 40–70 ng of dsRNA and were active. Mosquitoes were selected for dsRNA injection within 24 h after eclosion, as previously described. The mosquitoes were divided into three groups: wild-type (WT), GFP dsRNA-injected, and PGANT3 dsRNA-injected. Two days post-injection, when the dsRNA interference efficiency was expected to be at its peak (as indicated in Fig. 5B), the mosquitoes were placed in translucent cages and fed on blood meals as described earlier. After a strict 6-hours blood meal period, the mosquitoes were anaesthetized with carbon dioxide. We visually assessed which mosquitoes had taken a blood meal, categorizing them as the “blood group”. Subsequently, mosquitoes without obvious abdominal distention were dissected under a stereoscope, and those with blood in their mid-guts were also included in the “blood group”. The percentage of blood-fed or non-blood-fed mosquitoes relative to the total number of mosquitoes was used to calculate the blood-feeding rate. Triplicate experiments were performed to ensure reliability.

### Supplementary Information

**Additional file 1: Supplementary Table 1.** Heterozygosity of the selected species. Genome data were downloaded from SRA database. Heterozygosity was calculated using Jellyfish and GenomeScope.

**Additional file 2: Supplementary Table 2.** Completeness estimation of selected species. The genomes of each species were downloaded from NCBI. Genomic completeness was evaluated using BUSCO.

**Additional file 3: Supplementary Table 3.** Comparison of genome assembly using Canu, Wtdbg2, MECAT2 and Flye.

**Additional file 4: Supplementary Table 4.** Comparison of assembly statistics.

**Additional file 5: Supplementary Table 5.** Non-coding RNA in the newly assembly genome (AealbF3).

**Additional file 6: Supplementary Table 6.** The positively selected genes at the ancestral branch of hematophagous. Genes were annotated in KOBAS database, and those Chi-square test *p* value < 0.05 were showed.

**Additional file 7: Supplementary Figures 1-9.**

## Data Availability

All data relevant to study are included in the article or upload as online supplementary information. The datasets generated in the current study are available in the Genome Sequence Archive (GSA) at the National Genomics Data Center, Beijing Institute of Gemonics, Chinese Academy of Sciences/China National Center for Bioinformation (https://ngdc.cncb.ac.cn/gsub/). High quality assembled reads were obtained from GSA number: CRA012167, HiC data were obtained from GSA number: CRA012410. Transcriptome data of RNAi data were obtained from GSA number: CRA012298. Transcriptome data of different developmental stage were obtained from Bioproject: SRA245721 and PRJNA563095. Genome data of each species were downloaded from NCBI (https://www.ncbi.nlm.nih.gov/). Mitochondrial genome sequence was downloaded from Eukaryota mitochondrial genomes (https://www.ncbi.nlm.nih.gov/genomes/GenomesGroup.cgi?opt=organelle&taxid=2759).

## References

[1] Fang Y (2019). New strains of Japanese encephalitis virus circulating in Shanghai, China after a ten-year hiatus in local mosquito surveillance. Parasit Vectors.

[2] Tandina F (2018). Mosquitoes (Diptera: Culicidae) and mosquito-borne diseases in Mali, West Africa. Parasit Vectors.

[3] Rudolf I (2017). West Nile virus in overwintering mosquitoes, central Europe. Parasit Vectors.

[4] Xia D (2018). Photoperiodic diapause in a subtropical population of Aedes albopictus in Guangzhou, China: optimized field-laboratory-based study and statistical models for comprehensive characterization. Infect Dis Poverty.

[5] Piiroinen S (2013). Pre-invasion history and demography shape the genetic variation in the insecticide resistance-related acetylcholinesterase 2 gene in the invasive Colorado potato beetle. BMC Evol Biol.

[6] Kraemer MUG (2019). Past and future spread of the arbovirus vectors Aedes aegypti and Aedes albopictus. Nat Microbiol.

[7] Kraemer MU (2015). The global distribution of the arbovirus vectors aedes aegypti and ae. Albopictus. Elife.

[8] Cancrini G (2003). Aedes albopictus is a natural vector of dirofilaria immitis in Italy. Vet Parasitol.

[9] Younes L (2021). Dirofilaria immitis and dirofilaria repens in mosquitoes from Corsica Island. France Parasit Vectors.

[10] Guan D (2020). Identifying and removing haplotypic duplication in primary genome assemblies. Bioinformatics.

[11] Zhang X (2019). Assembly of allele-aware, chromosomal-scale autopolyploid genomes based on Hi-C data. Nat Plants.

[12] Chen XG (2015). Genome sequence of the Asian tiger mosquito, aedes albopictus, reveals insights into its biology, genetics, and evolution. Proc Natl Acad Sci U S A.

[13] Palatini U (2020). Improved reference genome of the arboviral vector Aedes albopictus. Genome Biol.

[14] Kumar A, Rai KS (1990). Intraspecific variation in nuclear DNA content among world populations of a mosquito, Aedes albopictus (Skuse). Theor Appl Genet.

[15] Matthews BJ (2018). Improved reference genome of Aedes aegypti informs arbovirus vector control. Nature.

[16] Zhang X (2020). Unzipping haplotypes in diploid and polyploid genomes. Comput Struct Biotechnol J.

[17] Xiao CL (2017). MECAT: fast mapping, error correction, and de novo assembly for single-molecule sequencing reads. Nat Methods.

[18] Roach MJ, Schmidt SA, Borneman AR (2018). Purge haplotigs: allelic contig reassignment for third-gen diploid genome assemblies. BMC Bioinformatics.

[19] Dudchenko O (2017). De novo assembly of the Aedes aegypti genome using Hi-C yields chromosome-length scaffolds. Science.

[20] Calle-Tobon A (2020). Surveillance of Zika virus in field-caught Aedes aegypti and Aedes albopictus suggests important role of male mosquitoes in viral populations maintenance in Medellin, Colombia. Infect Genet Evol.

[21] Monteiro VVS (2019). Aedes-Chikungunya Virus Interaction: key role of Vector midguts Microbiota and its saliva in the host infection. Front Microbiol.

[22] Wang Y (2018). Identification and evolution of olfactory genes in the small poplar longhorn beetle Saperda populnea. Comp Biochem Physiol Part D Genomics Proteom.

[23] Daniel EJP (2020). Ser and thr acceptor preferences of the GalNAc-Ts vary among isoenzymes to modulate mucin-type O-glycosylation. Glycobiology.

[24] Sakura R (2022). In vitro synthesis of mucin-type O-glycans using saccharide primers comprising GalNAc-Ser and GalNAc-Thr residues. Carbohydr Res.

[25] Staudacher E (2015). Mucin-Type O-Glycosylation in Invertebrates. Molecules.

[26] Tajadura-Ortega V (2021). O-linked mucin-type glycosylation regulates the transcriptional programme downstream of EGFR. Glycobiology.

[27] Burchell JM (2018). O-linked mucin-type glycosylation in breast cancer. Biochem Soc Trans.

[28] Dani N, Zhu H, Broadie K (2014). Two protein N-acetylgalactosaminyl transferases regulate synaptic plasticity by activity-dependent regulation of integrin signaling. J Neurosci.

[29] Zhang L, Zhang Y, Hagen KG (2008). A mucin-type O-glycosyltransferase modulates cell adhesion during Drosophila development. J Biol Chem.

[30] Zhang L, Ten Hagen KG (2010). Dissecting the biological role of mucin-type O-glycosylation using RNA interference in Drosophila cell culture. J Biol Chem.

[31] Alenou LD (2023). Burden of mosquito-borne diseases across rural versus urban areas in Cameroon between 2002 and 2021: prospective for community-oriented vector management approaches. Parasit Vectors.

[32] Baubichon-Cortay H (1989). Evidence for an O-glycan sialylation system in brain. Characterization of a beta-galactoside alpha 2,3-sialyltransferase from rat brain regulating the expression of an alpha-N-acetylgalactosaminide alpha 2,6-sialyltransferase activity. Eur J Biochem.

[33] Pan Y (2014). Podoplanin requires sialylated O-glycans for stable expression on lymphatic endothelial cells and for interaction with platelets. Blood.

[34] Chang YJ (2021). Endothelial-derived cardiovascular disease-related microRNAs elevated with prolonged sitting pattern among postmenopausal women. Sci Rep.

[35] Shi Y (2017). Mechanistic insights into precursor messenger RNA splicing by the spliceosome. Nat Rev Mol Cell Biol.

[36] Zhao Z (2022). Mosquito brains encode unique features of human odour to drive host seeking. Nature.

[37] Blumer M (2022). Gene losses in the common vampire bat illuminate molecular adaptations to blood feeding. Sci Adv.

[38] Achee NL (2019). Alternative strategies for mosquito-borne arbovirus control. PLoS Negl Trop Dis.

[39] Zhang L, Ten Hagen KG (2011). The cellular microenvironment and cell adhesion: a role for O-glycosylation. Biochem Soc Trans.

[40] Zhang L, Tran DT, Ten Hagen KG (2010). An O-glycosyltransferase promotes cell adhesion during development by influencing secretion of an extracellular matrix integrin ligand. J Biol Chem.

[41] Kong L (2023). Mosquito densovirus significantly reduces the vector susceptibility to dengue virus serotype 2 in Aedes albopictus mosquitoes (Diptera: Culicidae). Infect Dis Poverty.

[42] Marcais G, Kingsford C (2011). A fast, lock-free approach for efficient parallel counting of occurrences of k-mers. Bioinformatics.

[43] Ranallo-Benavidez TR, Jaron KS, Schatz MC (2020). GenomeScope 2.0 and Smudgeplot for reference-free profiling of polyploid genomes. Nat Commun.

[44] Goldberg DH (2009). Spike train analysis toolkit: enabling wider application of information-theoretic techniques to neurophysiology. Neuroinformatics.

[45] Kechin A (2017). cutPrimers: a New Tool for Accurate cutting of primers from reads of targeted next generation sequencing. J Comput Biol.

[46] Schmieder R, Edwards R (2011). Quality control and preprocessing of metagenomic datasets. Bioinformatics.

[47] Zhang H (2020). A useful tool to do the conformational sampling and trajectory analysis work for biomolecules. J Comput Chem.

[48] Koren S (2017). Canu: scalable and accurate long-read assembly via adaptive k-mer weighting and repeat separation. Genome Res.

[49] Ruan J, Li H (2020). Fast and accurate long-read assembly with wtdbg2. Nat Methods.

[50] Kolmogorov M (2019). Assembly of long, error-prone reads using repeat graphs. Nat Biotechnol.

[51] Simao FA (2015). BUSCO: assessing genome assembly and annotation completeness with single-copy orthologs. Bioinformatics.

[52] Li H, Durbin R (2009). Fast and accurate short read alignment with burrows-wheeler transform. Bioinformatics.

[53] Durand NC (2016). Juicer provides a one-click system for analyzing loop-resolution Hi-C experiments. Cell Syst.

[54] Durand NC (2016). Juicebox provides a visualization system for Hi-C contact maps with unlimited zoom. Cell Syst.

[55] Deschamps S (2018). A chromosome-scale assembly of the sorghum genome using nanopore sequencing and optical mapping. Nat Commun.

[56] Hu J (2020). NextPolish: a fast and efficient genome polishing tool for long-read assembly. Bioinformatics.

[57] Xu Z, Wang H (2007). LTR_FINDER: an efficient tool for the prediction of full-length LTR retrotransposons. Nucleic Acids Res.

[58] Flynn JM (2020). RepeatModeler2 for automated genomic discovery of transposable element families. Proc Natl Acad Sci U S A.

[59] Tarailo-Graovac M, Chen N (2009). Using RepeatMasker to identify repetitive elements in genomic sequences. Curr Protoc Bioinformatics.

[60] Bao W, Kojima KK, Kohany O (2015). Repbase Update, a database of repetitive elements in eukaryotic genomes. Mob DNA.

[61] Benson G (1999). Tandem repeats finder: a program to analyze DNA sequences. Nucleic Acids Res.

[62] Gardner PP (2011). Rfam: Wikipedia, clans and the decimal release. Nucleic Acids Res.

[63] Nawrocki EP, Kolbe DL, Eddy SR (2009). Infernal 1.0: inference of RNA alignments. Bioinformatics.

[64] Brown J, Pirrung M, McCue LA (2017). FQC Dashboard: integrates FastQC results into a web-based, interactive, and extensible FASTQ quality control tool. Bioinformatics.

[65] Bruna T (2021). BRAKER2: automatic eukaryotic genome annotation with geneMark-EP + and AUGUSTUS supported by a protein database. NAR Genom Bioinform.

[66] Fu L (2012). CD-HIT: accelerated for clustering the next-generation sequencing data. Bioinformatics.

[67] Buchfink B, Reuter K, Drost HG (2021). Sensitive protein alignments at tree-of-life scale using DIAMOND. Nat Methods.

[68] Chen C (2020). TBtools: an integrative toolkit developed for interactive analyses of big Biological Data. Mol Plant.

[69] Marcais G (2018). MUMmer4: a fast and versatile genome alignment system. PLoS Comput Biol.

[70] Edgar RC (2004). MUSCLE: multiple sequence alignment with high accuracy and high throughput. Nucleic Acids Res.

[71] Stamatakis A (2014). RAxML version 8: a tool for phylogenetic analysis and post-analysis of large phylogenies. Bioinformatics.

[72] Emms DM, Kelly S (2015). OrthoFinder: solving fundamental biases in whole genome comparisons dramatically improves orthogroup inference accuracy. Genome Biol.

[73] Kielbasa SM (2011). Adaptive seeds tame genomic sequence comparison. Genome Res.

[74] Wang D (2010). KaKs_Calculator 2.0: a toolkit incorporating gamma-series methods and sliding window strategies. Genomics Proteom Bioinf.

[75] Yang Z (2007). PAML 4: phylogenetic analysis by maximum likelihood. Mol Biol Evol.

[76] Finn RD (2016). The pfam protein families database: towards a more sustainable future. Nucleic Acids Res.

[77] Kim D (2013). TopHat2: accurate alignment of transcriptomes in the presence of insertions, deletions and gene fusions. Genome Biol.

[78] Liao Y, Smyth GK, Shi W (2014). featureCounts: an efficient general purpose program for assigning sequence reads to genomic features. Bioinformatics.

[79] Love MI, Huber W, Anders S (2014). Moderated estimation of Fold change and dispersion for RNA-seq data with DESeq2. Genome Biol.

[80] Langfelder P, Horvath S (2008). WGCNA: an R package for weighted correlation network analysis. BMC Bioinformatics.

[81] Shannon P (2003). Cytoscape: a software environment for integrated models of biomolecular interaction networks. Genome Res.

