# Supplementary material for: A high heterozygosity genome assembly of Aedes albopictus enables the discovery of the association of PGANT3 with blood-feeding behavior
Source: BMC Genomics. 2024 Apr 3;25:336. doi: 10.1186/s12864-024-10133-4 (PMC10993458; doi:10.1186/s12864-024-10133-4)
Supplement: Supplementary file 7 — Additional file 7: Supplementary Figures 1-9. [file 12864_2024_10133_MOESM7_ESM.pptx]

## Slide 1
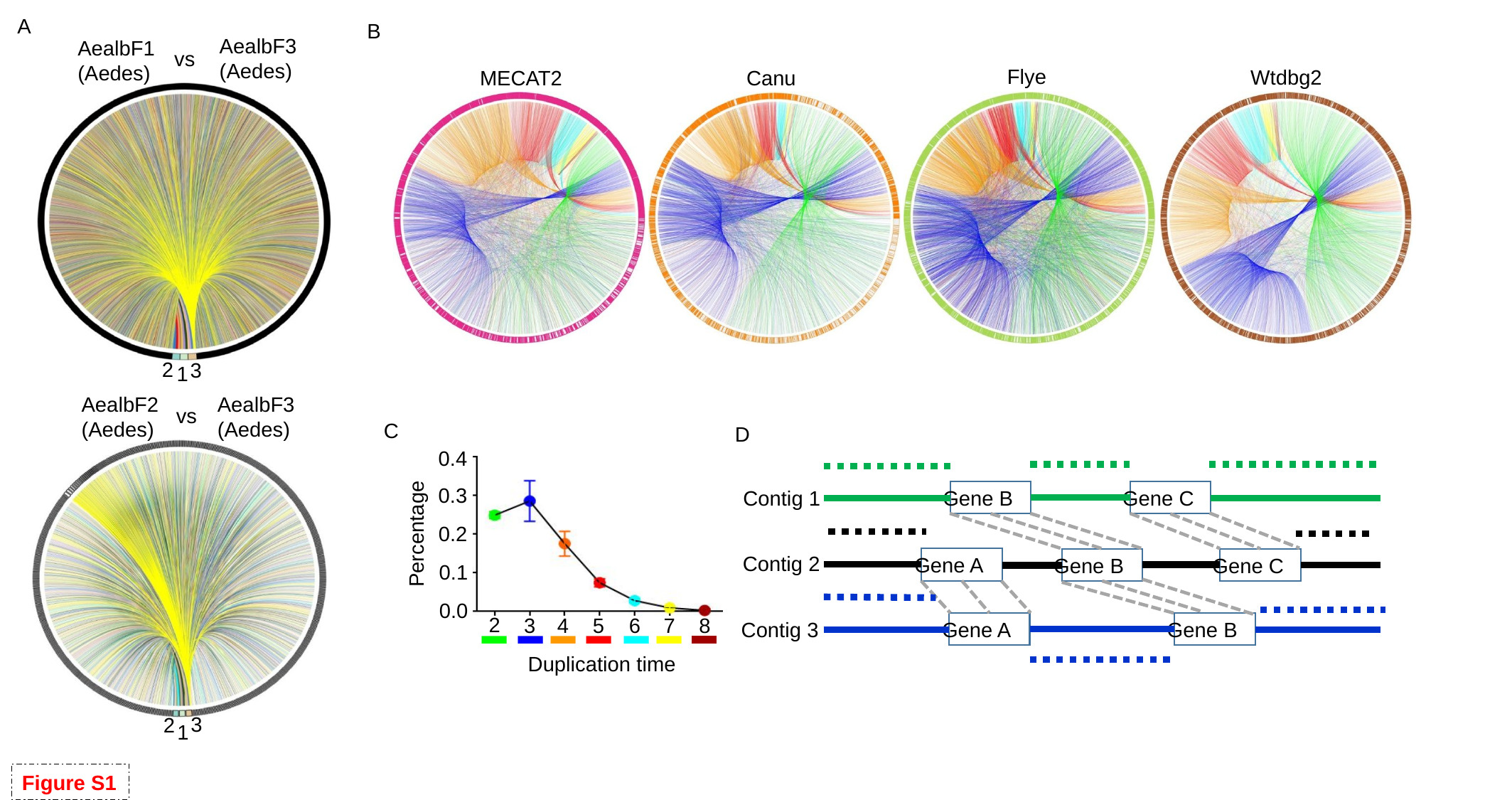

A
B
AealbF3
(Aedes)
AealbF1
(Aedes)
vs
2
3
1
Flye
Wtdbg2
MECAT2
Canu
AealbF2
(Aedes)
AealbF3
(Aedes)
vs
3
2
1
C
0.4
0.3
0.2
0.1
0.0
2
3
4
6
7
8
5
Duplication time
Percentage
D
Contig 1
Gene B
Gene C
Contig 2
Gene A
Gene B
Gene C
Gene A
Gene B
Contig 3
Figure S1

## Slide 2
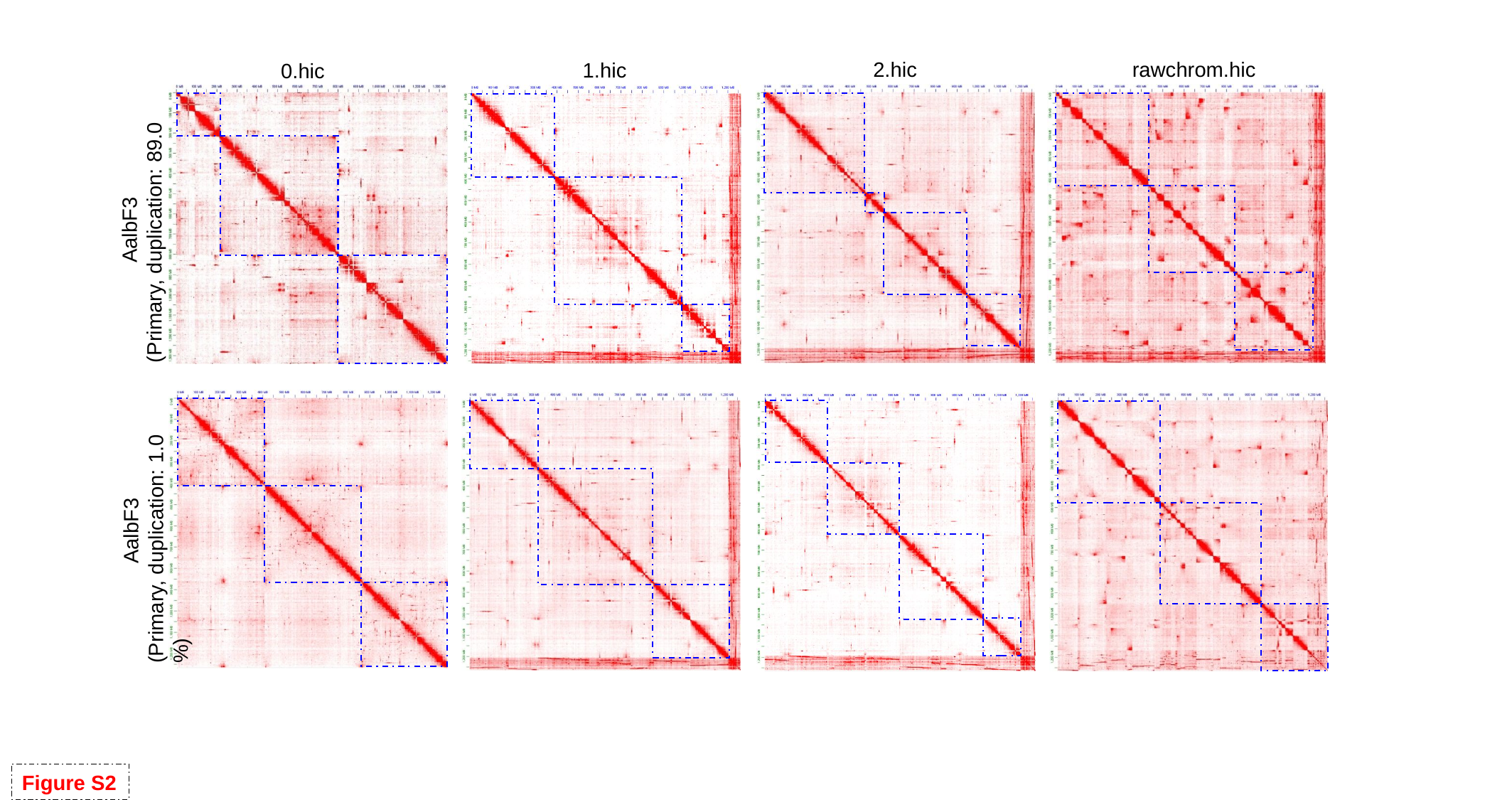

rawchrom.hic
2.hic
1.hic
0.hic
 AalbF3
(Primary, duplication: 89.0 %)
 AalbF3
(Primary, duplication: 1.0 %)
Figure S2

## Slide 3
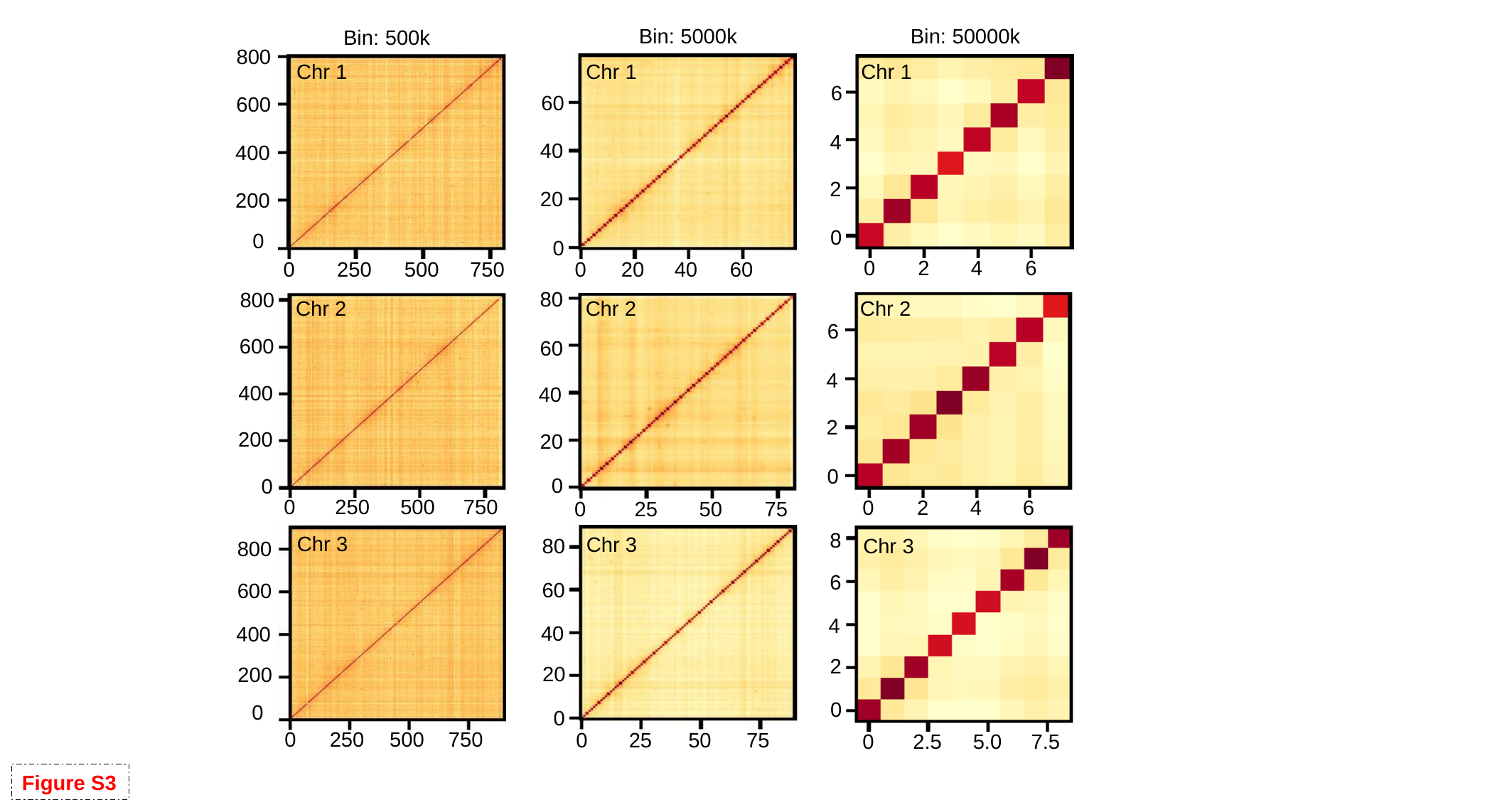

Bin: 5000k
Bin: 50000k
Bin: 500k
800
Chr 1
Chr 1
6
60
600
4
40
400
2
20
200
0
0
0
2
4
6
0
250
500
750
0
Chr 1
20
40
60
0
80
800
Chr 2
Chr 2
Chr 2
6
600
60
4
400
40
2
200
20
0
0
0
250
500
750
0
2
4
6
0
25
50
75
0
8
Chr 3
Chr 3
Chr 3
80
800
6
60
600
4
40
400
2
20
200
0
0
0
250
500
750
0
25
50
75
0
2.5
5.0
7.5
0
Figure S3

## Slide 4
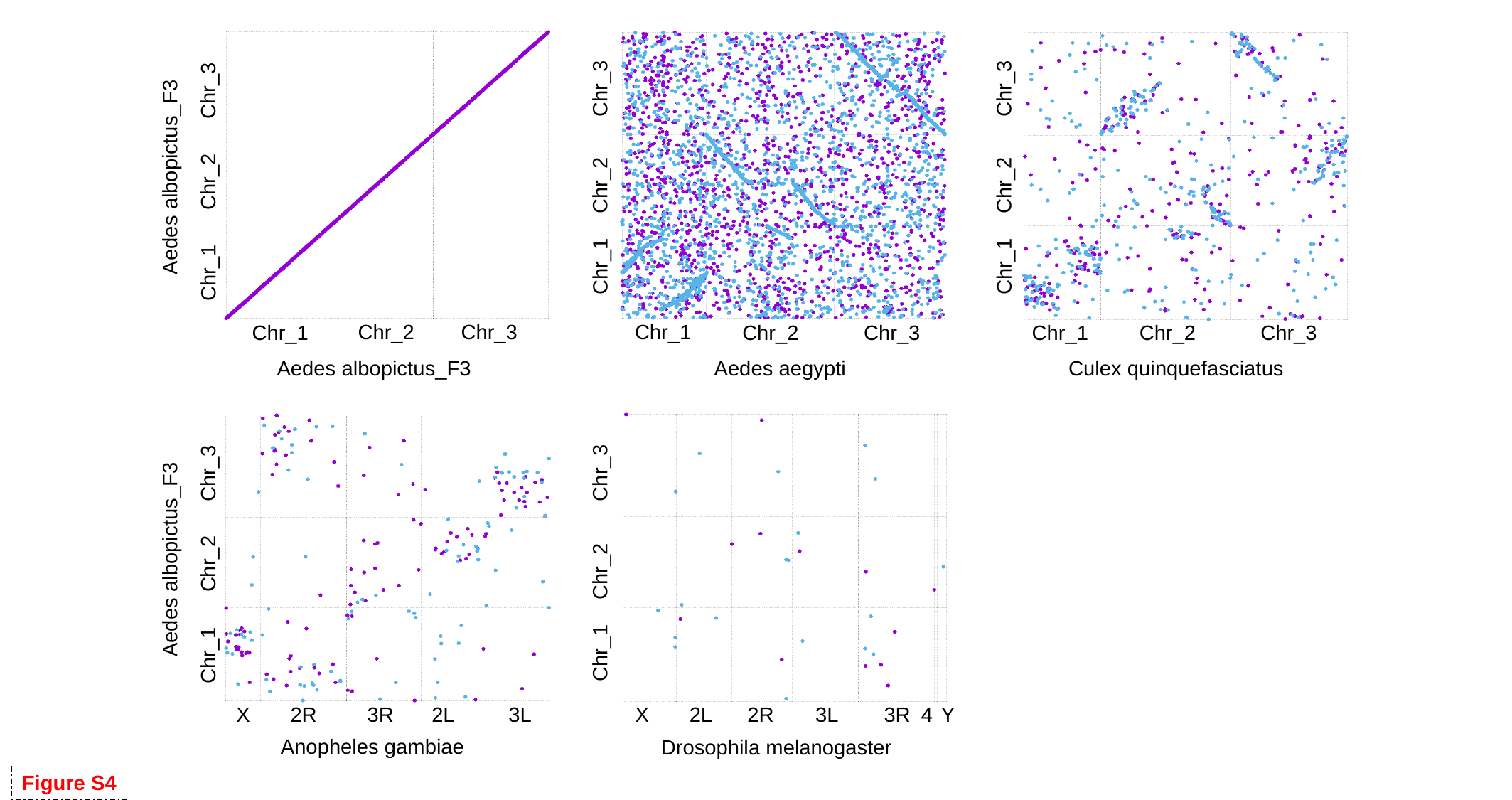

Chr_3
Chr_2
Chr_1
Chr_2
Chr_3
Chr_1
Aedes albopictus_F3
Aedes albopictus_F3
Chr_3
Chr_2
Chr_1
Chr_1
Chr_2
Chr_3
Aedes aegypti
Chr_3
Chr_2
Chr_1
Chr_1
Chr_2
Chr_3
Culex quinquefasciatus
Chr_3
Aedes albopictus_F3
Chr_2
Chr_1
X
2R
3R
2L
3L
Anopheles gambiae
Chr_3
Chr_2
Chr_1
X
2L
2R
3L
3R
4
Y
Drosophila melanogaster
Figure S4

## Slide 5
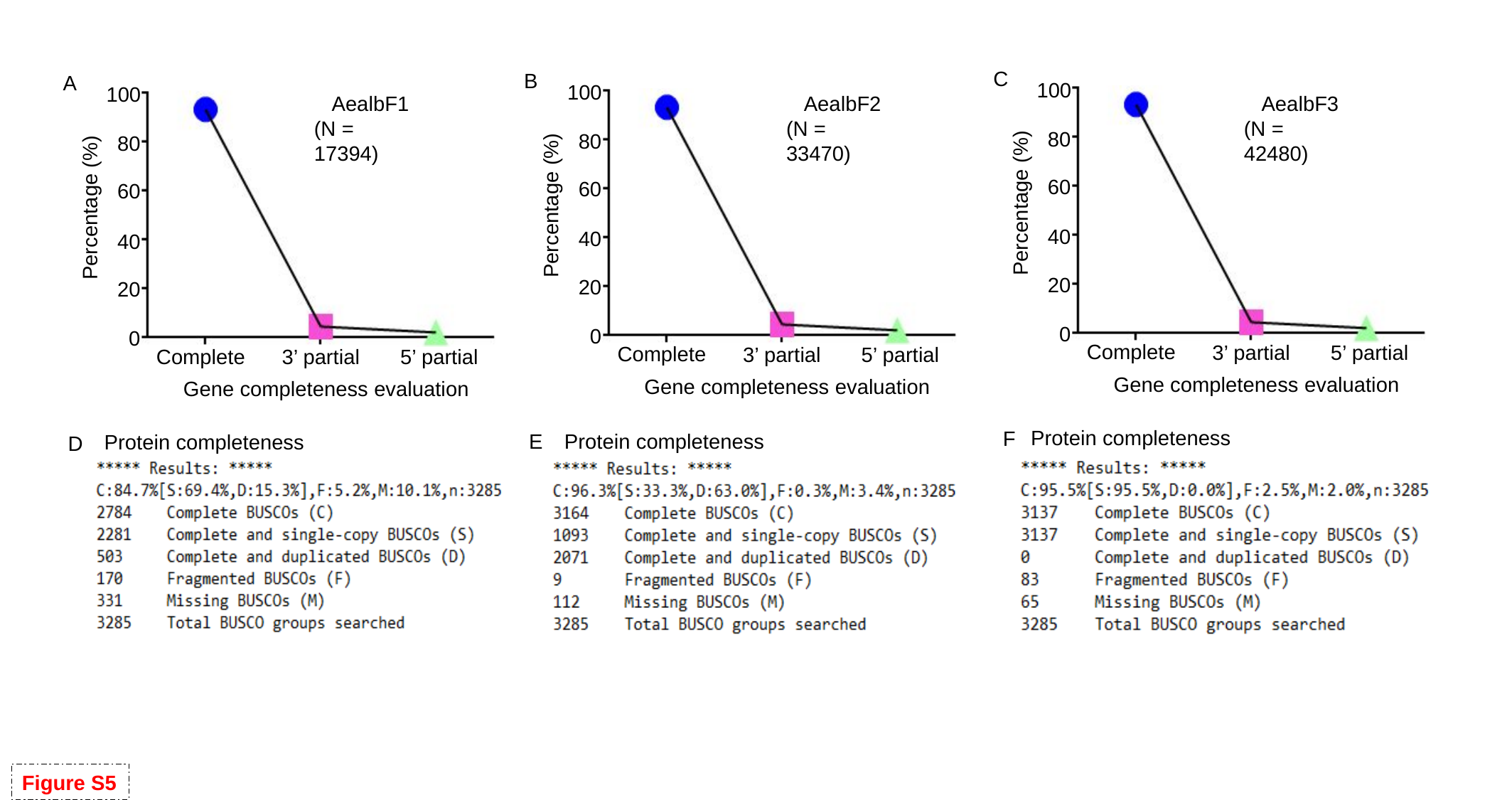

C
100
80
60
Percentage (%)
40
20
0
Complete
3’ partial
5’ partial
Gene completeness evaluation
 AealbF3
(N = 42480)
B
100
80
60
Percentage (%)
40
20
0
Complete
3’ partial
5’ partial
Gene completeness evaluation
 AealbF2
(N = 33470)
A
100
80
60
Percentage (%)
40
20
0
Complete
3’ partial
5’ partial
Gene completeness evaluation
 AealbF1
(N = 17394)
Protein completeness
F
E
Protein completeness
Protein completeness
D
Figure S5

## Slide 6
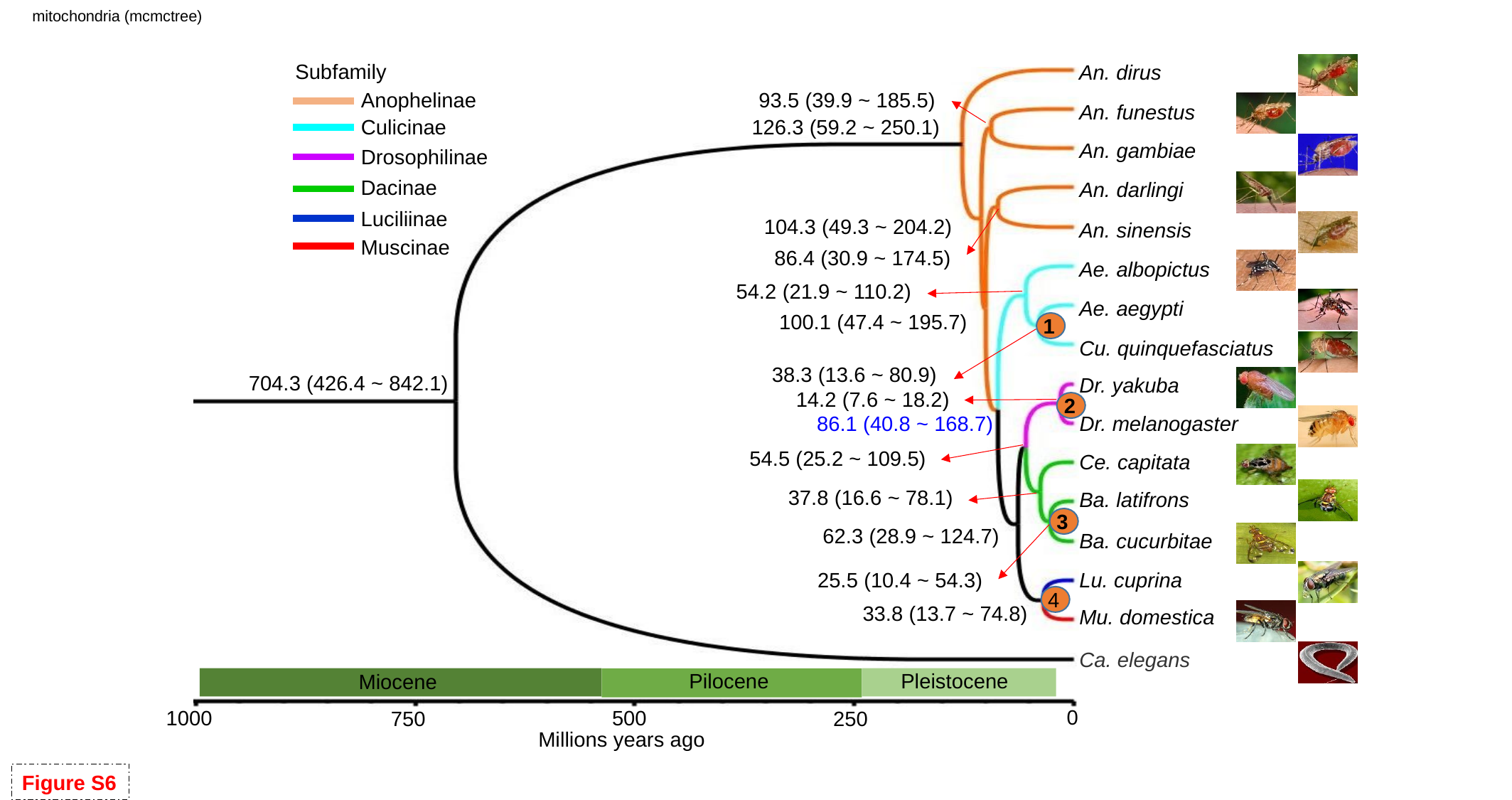

mitochondria (mcmctree)
Subfamily
Anophelinae
Culicinae
Drosophilinae
Dacinae
Luciliinae
Muscinae
An. dirus
93.5 (39.9 ~ 185.5)
An. funestus
126.3 (59.2 ~ 250.1)
An. gambiae
An. darlingi
104.3 (49.3 ~ 204.2)
An. sinensis
86.4 (30.9 ~ 174.5)
Ae. albopictus
54.2 (21.9 ~ 110.2)
Ae. aegypti
100.1 (47.4 ~ 195.7)
Cu. quinquefasciatus
38.3 (13.6 ~ 80.9)
704.3 (426.4 ~ 842.1)
Dr. yakuba
14.2 (7.6 ~ 18.2)
Dr. melanogaster
86.1 (40.8 ~ 168.7)
54.5 (25.2 ~ 109.5)
Ce. capitata
37.8 (16.6 ~ 78.1)
Ba. latifrons
62.3 (28.9 ~ 124.7)
Ba. cucurbitae
Lu. cuprina
25.5 (10.4 ~ 54.3)
33.8 (13.7 ~ 74.8)
Mu. domestica
Ca. elegans
Pilocene
Pleistocene
Miocene
0
500
1000
750
250
Millions years ago
1
2
3
4
Figure S6

## Slide 7
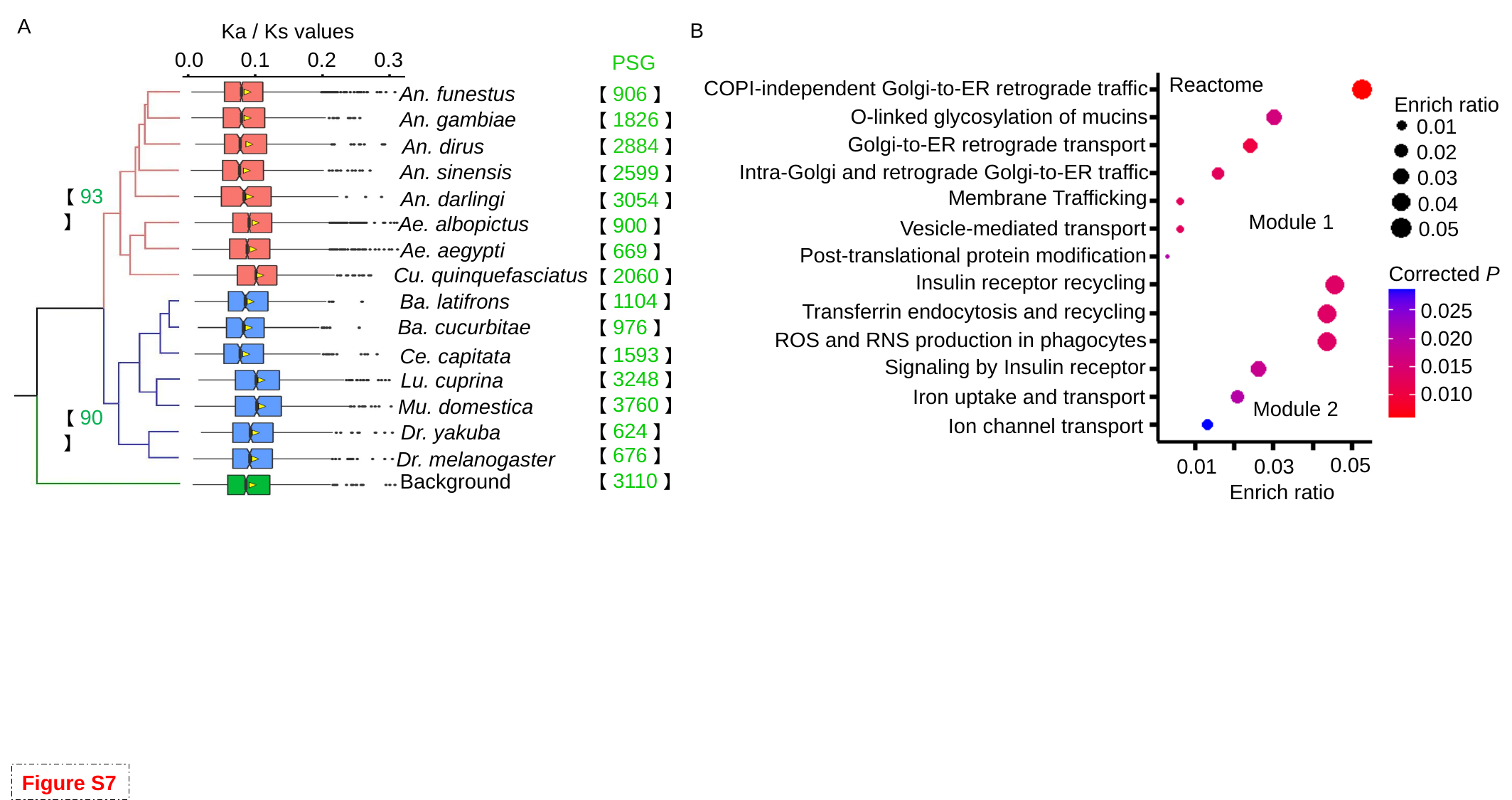

A
B
Ka / Ks values
0.1
0.2
0.3
0.0
PSG
An. funestus
An. gambiae
An. dirus
An. sinensis
An. darlingi
Ae. albopictus
Ae. aegypti
Cu. quinquefasciatus
Ba. latifrons
Ba. cucurbitae
Ce. capitata
Lu. cuprina
Mu. domestica
Dr. yakuba
Dr. melanogaster
Background
【906】
【1826】
【2884】
【2599】
【3054】
【900】
【669】
【2060】
【1104】
【976】
【1593】
【3248】
【3760】
【624】
【676】
【3110】
【93】
【90】
Reactome
COPI-independent Golgi-to-ER retrograde traffic
0.05
0.03
0.01
Enrich ratio
Enrich ratio
0.01
0.02
0.03
0.04
0.05
O-linked glycosylation of mucins
Golgi-to-ER retrograde transport
Intra-Golgi and retrograde Golgi-to-ER traffic
Membrane Trafficking
Module 1
Vesicle-mediated transport
Post-translational protein modification
Corrected P
0.025
0.020
0.015
0.010
Insulin receptor recycling
Transferrin endocytosis and recycling
ROS and RNS production in phagocytes
Signaling by Insulin receptor
Iron uptake and transport
Module 2
Ion channel transport
Figure S7

## Slide 8
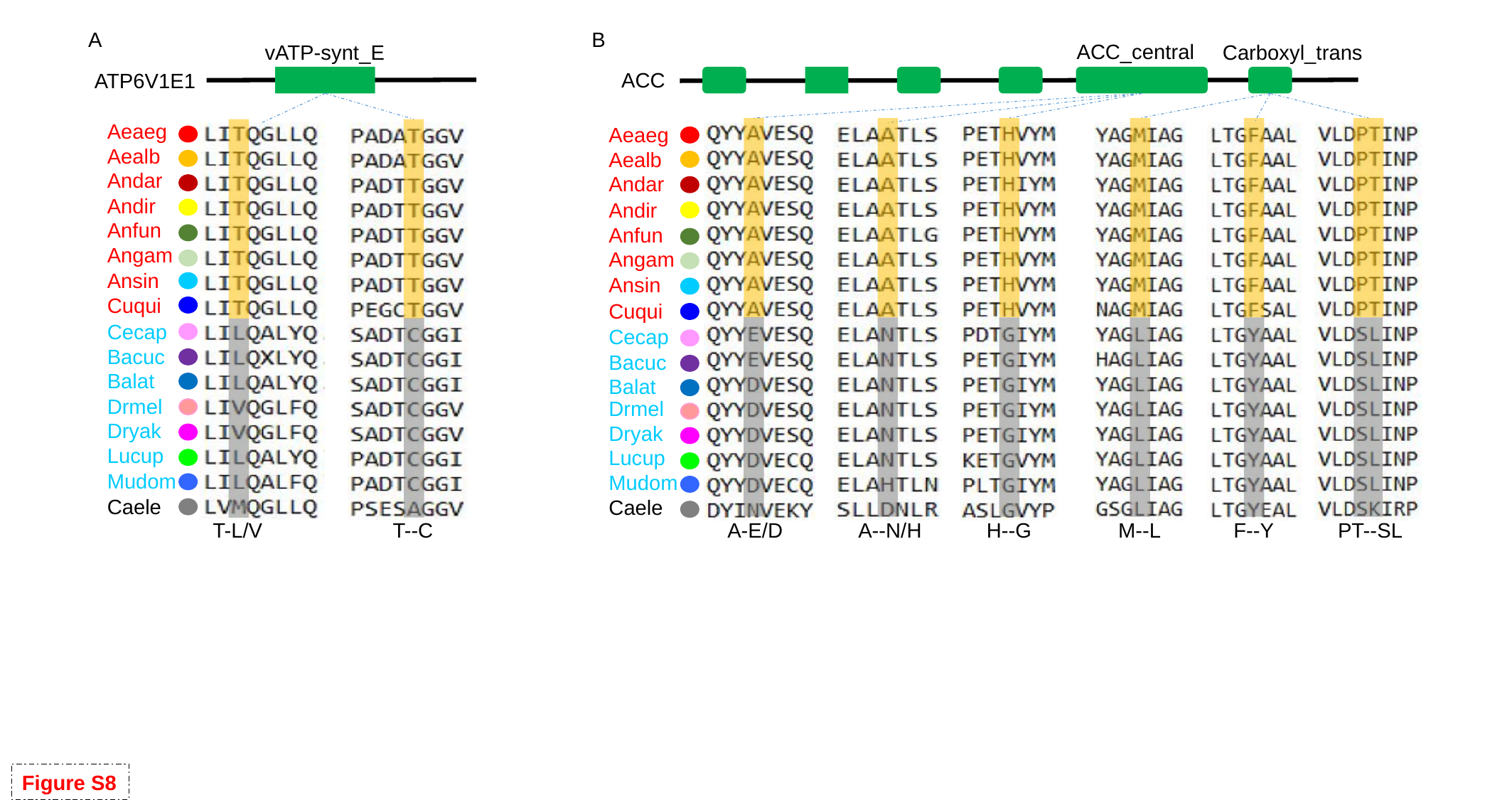

B
ACC_central
Carboxyl_trans
ACC
Aeaeg
Aealb
Andar
Andir
Anfun
Angam
Ansin
Cuqui
Cecap
Bacuc
Balat
Drmel
Dryak
Lucup
Mudom
Caele
A-E/D
A--N/H
H--G
M--L
F--Y
PT--SL
A
vATP-synt_E
ATP6V1E1
T-L/V
T--C
Aeaeg
Aealb
Andar
Andir
Anfun
Angam
Ansin
Cuqui
Cecap
Bacuc
Balat
Drmel
Dryak
Lucup
Mudom
Caele
Figure S8

## Slide 9
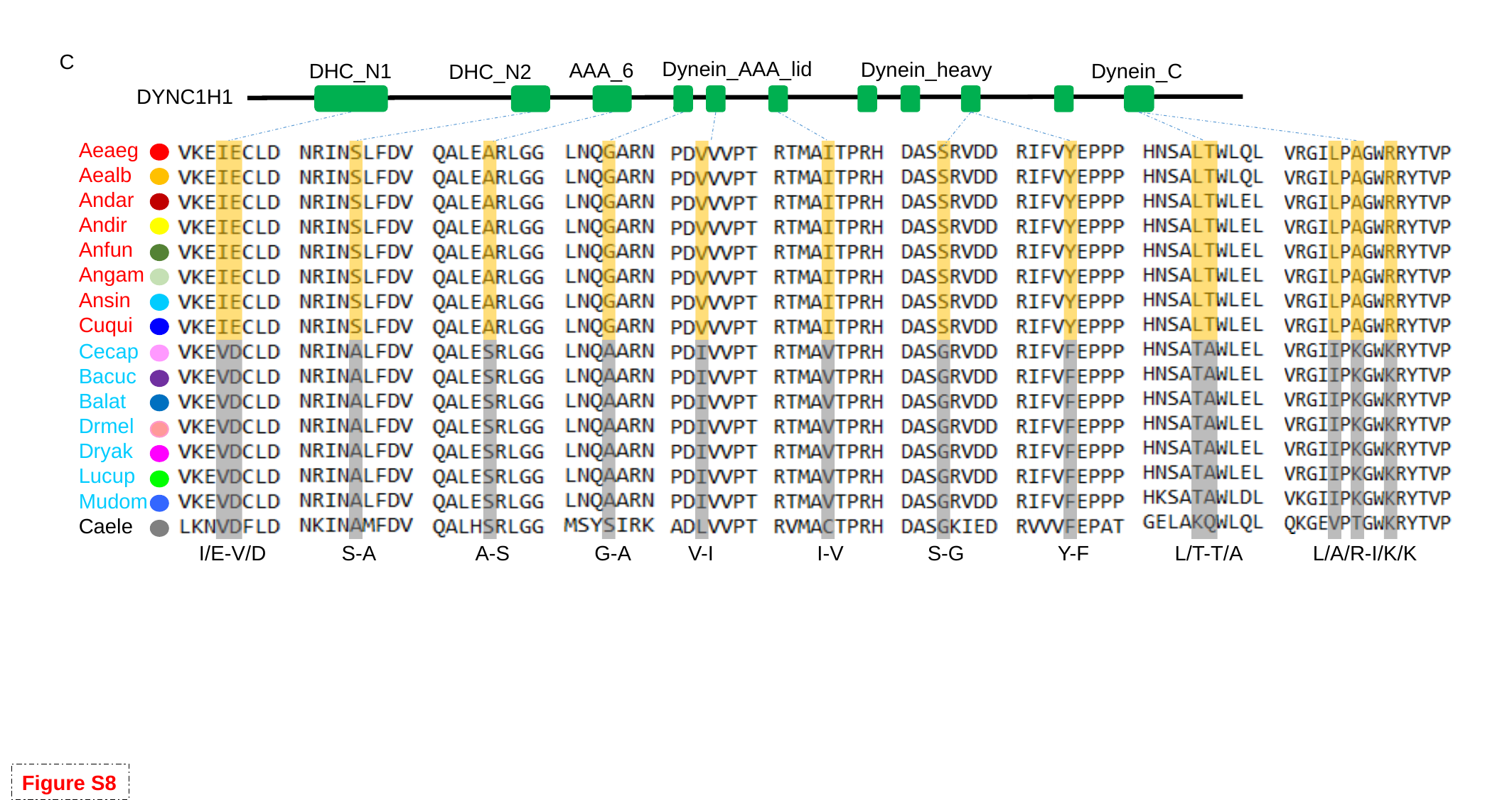

C
Dynein_AAA_lid
Dynein_heavy
AAA_6
DHC_N1
Dynein_C
DHC_N2
DYNC1H1
Aeaeg
Aealb
Andar
Andir
Anfun
Angam
Ansin
Cuqui
Cecap
Bacuc
Balat
Drmel
Dryak
Lucup
Mudom
Caele
G-A
V-I
I/E-V/D
S-A
A-S
I-V
S-G
Y-F
L/T-T/A
L/A/R-I/K/K
Figure S8

## Slide 10
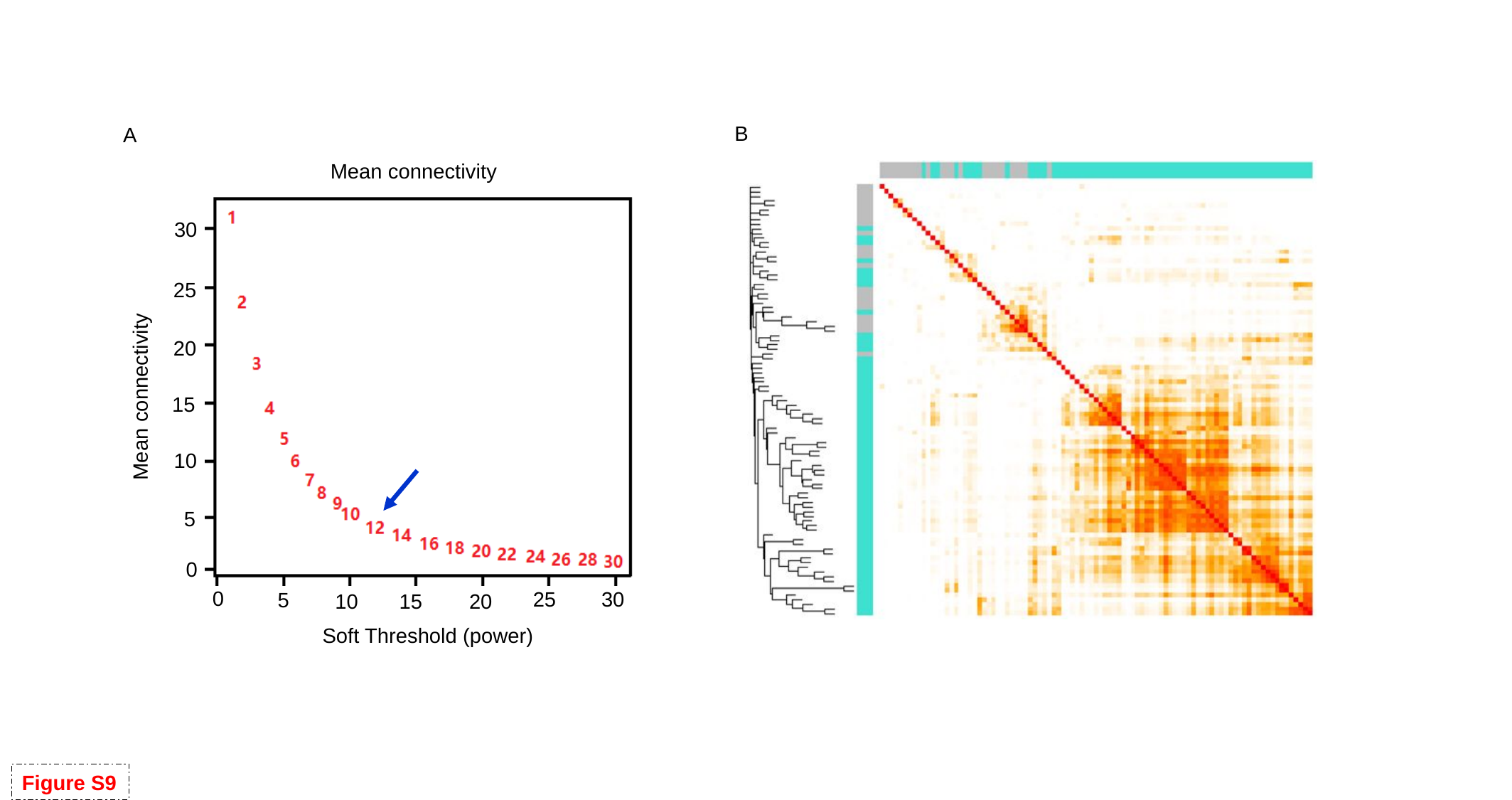

B
A
Mean connectivity
30
25
20
Mean connectivity
15
10
5
0
0
25
30
5
15
10
20
Soft Threshold (power)
Figure S9
